# Challenging social media threats using collective well-being-aware recommendation algorithms and an educational virtual companion

**DOI:** 10.3389/frai.2022.654930

**Published:** 2023-01-09

**Authors:** Dimitri Ognibene, Rodrigo Wilkens, Davide Taibi, Davinia Hernández-Leo, Udo Kruschwitz, Gregor Donabauer, Emily Theophilou, Francesco Lomonaco, Sathya Bursic, Rene Alejandro Lobo, J. Roberto Sánchez-Reina, Lidia Scifo, Veronica Schwarze, Johanna Börsting, Ulrich Hoppe, Farbod Aprin, Nils Malzahn, Sabrina Eimler

**Affiliations:** ^1^Department of Psychology, University of Milano-Bicocca, Milan, Italy; ^2^Faculty of Science and Health, School of Computer Science and Electronic Engineering, University of Essex, Colchester, United Kingdom; ^3^Cental, Institut Langage et Communication (IL&C), Université catholique de Louvain (UCLouvain), Ottignies-Louvain-la-Neuve, Belgium; ^4^Institute for Education Technology, National Research Council of Italy, Palermo, Italy; ^5^Department of Information and Communication Technologies, Pompeu Fabra University, Barcelona, Spain; ^6^Faculty of Information Science, University of Regensburg, Regensburg, Germany; ^7^Institute of Computer Science, Ruhr West University of Applied Science, Bottrop, Germany; ^8^Rhein-Ruhr Institut für Angewandte Systeminnovation, Duisburg, Germany

**Keywords:** collective well-being, recommender systems, social media, virtual companion, social media threats, hierarchical reinforcement learning

## Abstract

Social media have become an integral part of our lives, expanding our interlinking capabilities to new levels. There is plenty to be said about their positive effects. On the other hand, however, some serious negative implications of social media have been repeatedly highlighted in recent years, pointing at various threats to society and its more vulnerable members, such as teenagers, in particular, ranging from much-discussed problems such as digital addiction and polarization to manipulative influences of algorithms and further to more teenager-specific issues (e.g., body stereotyping). The impact of social media—both at an individual and societal level—is characterized by the complex interplay between the users' interactions and the intelligent components of the platform. Thus, users' understanding of social media mechanisms plays a determinant role. We thus propose a theoretical framework based on an adaptive “*Social Media Virtual Companion*” for educating and supporting an entire community, teenage students, to interact in social media environments in order to achieve desirable conditions, defined in terms of a community-specific and participatory designed measure of Collective Well-Being (CWB). This Companion combines automatic processing with expert intervention and guidance. The virtual Companion will be powered by a *Recommender System* (*CWB-RS*) that will optimize a *CWB* metric instead of engagement or platform profit, which currently largely drives recommender systems thereby disregarding any societal collateral effect. CWB-RS will optimize CWB both in the short term by balancing the level of social media threats the users are exposed to, and in the long term by adopting an *Intelligent Tutor System* role and enabling adaptive and personalized sequencing of playful learning activities. We put an emphasis on *experts* and *educators* in the *educationally managed social media community* of the Companion. They play five key roles: (a) use the Companion in classroom-based educational activities; (b) guide the definition of the CWB; (c) provide a hierarchical structure of learning strategies, objectives and activities that will support and contain the adaptive sequencing algorithms of the CWB-RS based on hierarchical reinforcement learning; (d) act as moderators of direct conflicts between the members of the community; and, finally, (e) monitor and address ethical and educational issues that are beyond the intelligent agent's competence and control. This framework offers a possible approach to understanding how to design social media systems and embedded educational interventions that favor a more healthy and positive society. Preliminary results on the performance of the Companion's components and studies of the educational and psychological underlying principles are presented.

## 1. Introduction

Social media (SM) have become an integral part of our everyday lives. Looking at the field more broadly, the freedom to post whatever someone judges useful has been described as nothing less than a shift in the communication paradigm (Baeza-Yates and Ribeiro-Neto, 1999), or in other words, *the freedom to publish* marks the birth of a new era altogether (Baeza-Yates and Ribeiro-Neto, 2010). There is ample evidence of positive effects of SM that goes beyond just-in-time connectivity with a network of friends and like-minded people, including, but not limited to, improved relationship maintenance (Ellison et al., 2014), increased intimacy (Jiang et al., 2011), reduced loneliness (Khosravi et al., 2016; Ryan et al., 2017), and reduced depression (Grieve et al., 2013). It has become a highly accessible and increasingly popular means of sharing content and immediately re-sharing others' content. Supported by personalizing recommendation algorithms, which suggest content and contacts, SM allows information of any quality to spread at an exponentially faster rate than the traditional “word of mouth” (Murthy, 2012; Webb et al., 2016). However, far from creating a global space for mutual understanding, truthful, and objective information, the large-scale growth of SM has also fostered negative social phenomena, e.g., (cyber)bullying to pick just one (Cowie, 2013; Mladenović et al., 2021), that only existed on a limited scale and slow pace before the digital revolution. These issues are escalated by impulsive, alienating and excessive usage that can be associated with digital addiction (Almourad et al., 2020). These phenomena, enabled by the rapid spread of information on SM can affect the well-being of more vulnerable members of our society, such as teenagers, in particular (Talwar et al., 2014; Ozimek et al., 2017; Gao et al., 2020). Ever since the Cambridge Analytica scandal (Isaak and Hanna, 2018), we have become more sensitive to the negative implications of social media. One might go as far as to suggest that SM may have become so dangerous that we would be in a better place without them, but that is clearly an unrealistic idea.

It can be argued that the impact of online experience, especially in SM, intrinsically depends on the mutual attitudes and interactions between the members of the community (Jones and Mitchell, 2016) and their interplay with the intelligent components of the platforms. This calls for a holistic approach that on one side provides *educational interventions* supporting users in understanding the impact of their actions on the experience of the other members of the community (Jones and Mitchell, 2016; Xu et al., 2019; Taibi et al., 2022) and their role in the *Collective well-being of their social media community (CWB)* (Ahn and Shin, 2013; Roy et al., 2018; Allcott et al., 2020). CWB operationally combines the different aspects of what a community considers its “desirable condition,” while also crucially considering individual differences and conflicting interests. Moreover, the lack of users' “*new media literacy”* (Scolari et al., 2018) (i.e., understanding of social media mechanisms) has a strong role in escalating SM threats. For example, a study with middle-school students found that more than 80% of them believed that the “sponsored content” articles shown to them were true stories (Wineburg et al., 2016). On the other side a multifaceted approach needs to provide technological support to reduce the strain cognitive resources of social media users. An important question is how this can be realized considering the complexity of the involved phenomena, the diverse attitudes and interests of the users, the cost of an intervention with a coverage and impact comparable to that of social media.

With this motivation, in this paper, we articulate a framework for educating teenagers in their interaction with SM and synergetically improve and support their experience based on a “*Social Media Virtual Companion*.” Inside an external SM platform, it will create an *educationally managed social media community* where playful learning activities and healthy content will be integrated into participants' SM experience. Educational goals and interventions will be designed by experts and educators, e.g., to raise awareness about potential threats and to show alternative healthy interactions. To select the most suitable content and effective interventions based on experts' and educators' designs, the companion will incorporate functions of an Intelligent Tutor System (ITS).

**Figure 1:**
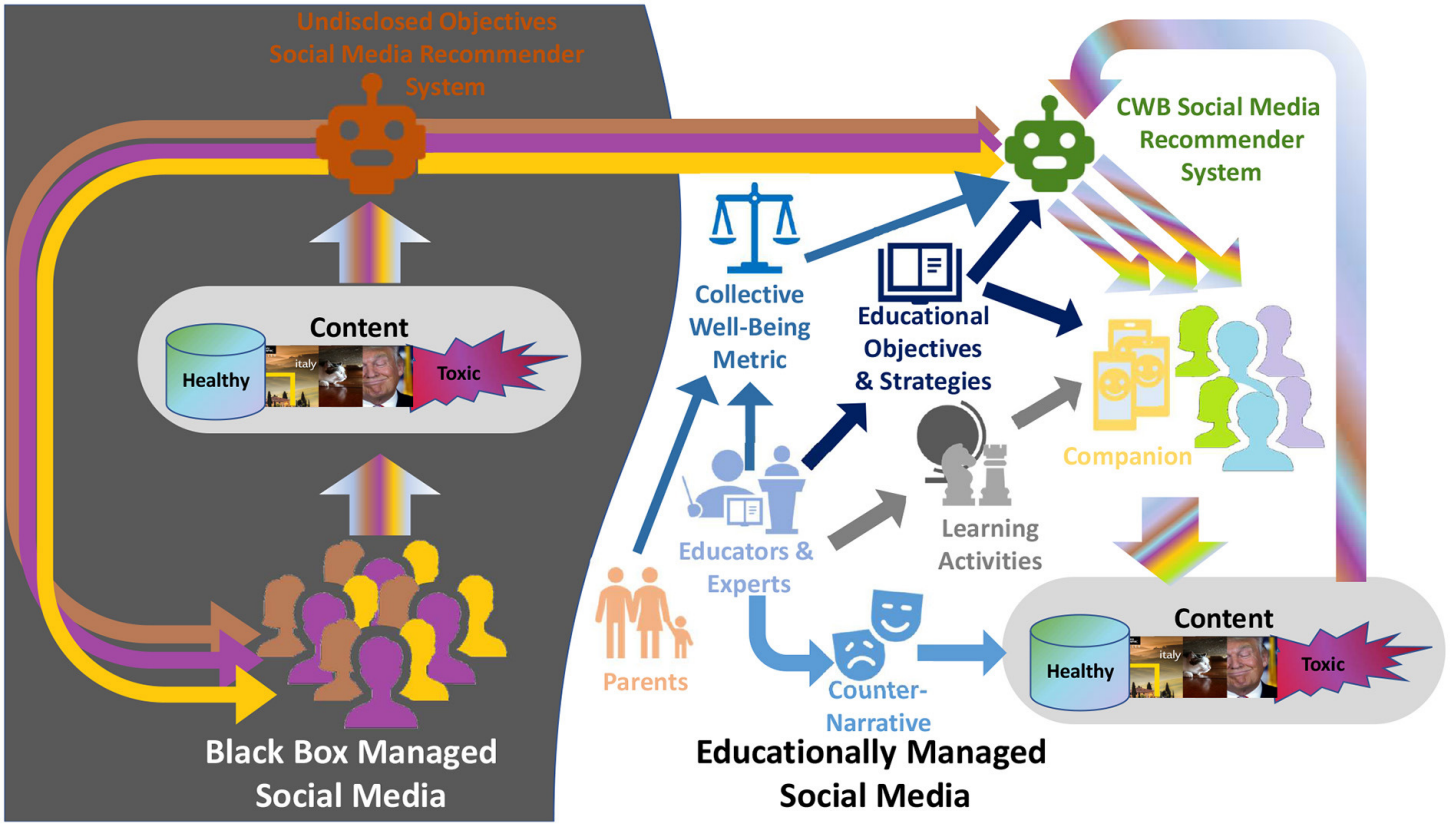
The virtual *Social Media Companion* enables continue educational and interaction support for a community of students with the involvement of educators. This generates an *Educationally Managed Social Media Community* whose Collective Well-Being is actively improved by the CWB-RS powering the Companion under the guidance of the educational objectives and strategies provided by the educators.

Due to the cognitively burdening and overloading information flow of the current SM platform (see Section 2.4 and Weng et al., 2012; Kramer et al., 2014; Lee et al., 2019; Almourad et al., 2020), the Companion will also have to balance and ignore engagement-driven external platform recommendations to target for a fairer and healthier objective (Rastegarpanah et al., 2019). The community of users of the SM platform is both the producer and consumer of SM content. We affirm that the objective pursued by SM algorithms should be closer to the community's needs than those of the SM platform. As the CWB reflects the global impact of MS on the condition of the individual and the community, we propose that a suitable objective is a measure that formalizes community-specific and participatory designed CWB expanded with student-specific educational objectives. This shifts to a *CWB* metric evaluated directly on the Companion and used as an optimization target by its integrated recommendation engine (*CWB-RS)*, which will allow the support and educational management of the local social media community (see Figure 1).

This framework can be seen as a top-down vision that combines education and technology complements, integrates and helps to balance the diverse efforts targeting specific SM issues. We think that social media phenomena' complexity, their intrinsically interlinked nature, and their impact on our society demand the production and discussion of such overarching views in the scientific community. Furthermore, our framework, by proposing educational social media that can be separated or linked with the main social media, would improve the problems that arise from platform-enforced restrictions that hinder experimentation and analysis, especially in extended longitudinal studies. The data collected could be the first step to enabling the definition of adequate regulations and revising SM platform designs to improve their impact on society.

In the next section, a concise overview of the SM threats is presented. In Section 3, we present the educational Companion approach for the increase of digital literacy and the enhancement of CWB. In Section 4, we discuss the CWB metrics. The CWB-RS is presented in Section 5 while, in Section 6, we present a use case exemplifying the interaction of the SM users with the Companion and CWB-RS. In Section 7.1 we present the current advances of this line of research.

## 2. Social media threats

With the advent of social media, the speed and number of interactions escalated beyond users' ability to monitor and understand their impact. This resulted in challenging threats with a broad range and variability over time, compounded by crucial ethical and practical issues, like preserving freedom of speech and allowing users to be collectively satisfied while dealing with the conflicts generated by their different opinions and contrasting interests. These are magnified by the complex dynamics of information on social media due to the interaction between myriads of users and intelligent artificial systems.

Critical cases are the pervasive diffusion of fake news and biased content and the growing trend of hate practices. Indeed, hate propagators were among the early adopters of the Internet (Schafer, 2002; Gerstenfeld et al., 2003; Chan et al., 2016). Even though SM platforms presenting policies against hate speech and discrimination, these new media have been shown to be powerful tools to reach new audiences and spread racist propaganda and incite violence offline. This gave rise to concern of several human rights associations about the platforms' usage to spread all forms of discrimination^1^ (Chris Hale, 2012; Bliuc et al., 2018).

The social media threats can be broadly classified into three categories: 1. content; 2. algorithmic; network, and attacks; and 3. dynamics. However, sharply separating these types of threats is not trivial as they strongly interact and mutually reinforce while often leveraging on several cognitive aspects and limits of the users. In the rest of this section, we briefly discuss SM threats, and we present in **Table 2** a list of examples per category. We focus on threats that specifically affect the vulnerable population of teenagers and the related threats, such as bullying (Talwar et al., 2014; Mladenović et al., 2021; Fulantelli et al., 2022), addiction (Tariq et al., 2012; Shensa et al., 2017), body stereotypes, and others

### 2.1. Content-based social media threats

The content-based threats are common to classical media, but specific issues thrive on the web and social media in particular. Examples of content-based threats include toxic content (Kozyreva et al., 2020), fake news/disinformation (de Cock Buning, 2018), beauty stereotypes (Verrastro et al., 2020), and bullying (Grigg, 2010). Given the importance of these threats, various research is focused on the development of dedicated detection systems as discussed in Section 5.4.

### 2.2. Algorithmic social media threats

The SM algorithms may create additional threats. For example, the selective exposure of digital media users to news sources (Schmidt et al., 2017), risks creating a permanent distorting state of isolation from different ideas and perspectives, i.e., “filter bubbles” (Nikolov et al., 2015; Geschke et al., 2019), and form closed-group polarized social structures, i.e., “echo chambers” (Del Vicario et al., 2016; Gillani et al., 2018). Another undesired network condition is gerrymandering (Stewart et al., 2019), where users are exposed to unbalanced neighborhood configurations.

### 2.3. Social media dynamics induced threats

The social media dynamics induced by the extended and fast-paced interaction between their algorithms, common social tendencies, and stakeholders' interests may also be a source of threats (Anderson and McLaren, 2012; Milano et al., 2021). These factors may escalate the acceptance of toxic beliefs (Neubaum and Krämer, 2017; Stewart et al., 2019), make social media users' opinions susceptible to phenomena such as the diffusion of hateful content, and induce violent outbreaks of fake news on a large scale (Del Vicario et al., 2016; Webb et al., 2016).

### 2.4. Social media cognitive and socio-emotional threats

While many studies that analyze the mechanisms of content propagation in social media exist, how to model the effects of users' emotional and cognitive states or traits on the propagating malicious content is unclear, especially in light of the significant contribution of their cognitive limits (Weng et al., 2012; Allcott and Gentzkow, 2017; Pennycook and Rand, 2018). Important cognitive factors are users' limited attention and error-prone information processing (Weng et al., 2012) that may be worsened by the emotional features of the messages (Kramer et al., 2014; Brady et al., 2017). Moreover, the lack of non-verbal communication and limited social presence (Gunawardena, 1995; Rourke et al., 1999; Mehari et al., 2014) often exasperates carelessness and misbehaviors, as the users perceive themselves as anonymous (Diener et al., 1980; Postmes and Spears, 1998), do not feel judged or exposed (Whittaker and Kowalski, 2015) and deindividualize themselves and other users (Lowry et al., 2016).

Over time, users' behaviors can deteriorate and show highly impulsive and addictive traits (Kuss and Griffiths, 2011). Indeed, social media usage presents many neurocognitive characteristics (e.g., the presence of impulsivity) typical of more established forms of pharmacological and behavioral addictions (Lee et al., 2019). This recently recognized threat, named Digital Addiction (DA) (Lavenia, 2012; Nakayama and Higuchi, 2015; Almourad et al., 2020), has several harmful consequences, such as unconscious and hasty actions (Ali et al., 2015; Alrobai et al., 2016). Some of them are especially relevant for teenagers affecting their school performance and mood (Aboujaoude et al., 2006). In the last few years, it emerged that recognizing addiction to social media cannot be based only on the “connection time” criterion but also on how people behave (Taymur et al., 2016; Musetti and Corsano, 2018). Like in the other behavioral addictions, a crucial role may be played by the environment structure (Kurth-Nelson and Redish, 2009; Ognibene et al., 2019), more than by biochemical failures of the decision system (Lim et al., 2019). Indeed, many, if not all, aspects of social media environments are under the control of the recommender systems, which may help reduce the condition with specific strategies, such as higher delays for more impulsive users as well as detecting and curbing its triggers, e.g., feelings of Fear of Missing Out (Alutaybi et al., 2019).

### 2.5. Limited social media literacy

Finally, the lack of digital literacy, common among teenagers (Meyers et al., 2013), can strongly contribute to other threats escalation, for example by favoring the spread of content-based threats and engaging in toxic dynamics (Wineburg et al., 2016). Teenagers also show over-reliance on algorithmic recommendations and a lack of awareness of the unwitting use of toxic content. Thus, reducing their ability to make choices and increasingly deviating toward dangerous behaviors (Walker, 2016; Banker and Khetani, 2019).

This diverse set of phenomena and threats, the latter in particular, motivates our educational approach combining educational methods to rise digital citizenship and new median literacy while supporting the user with a smart companion that can also counter the cognitive burden of interacting with social media.

## 3. Educational social media companion

Social media have been shown to contribute to our collective well-being enhancing our levels of social connectivity. However, our well-being, and in particular teenagers' one, is vulnerable to social media threats, such as exposure to many types of unwanted or toxic content (Costello et al., 2019; Mladenović et al., 2021). Increasing social media users' digital literacy (Fedorov, 2015) and citizenship (Jones and Mitchell, 2016; Xu et al., 2019) may counter most SM threats that thrive due to users' lack of awareness and over-reliance on algorithmic recommendations (Meyers et al., 2013; Walker, 2016; Banker and Khetani, 2019).

The traditional media literacy approaches were based on the idea that media had adverse effects on children. Therefore, it was necessary to “immunize” young people so they can resist such negative influence. As the media ecosystem evolved, so did media literacy. It soon included a paradigm shift toward education and risk prevention concerning the web, video games, social networks and mobile devices. Recently, new concepts have been developed to name these new forms of literacy, from “digital literacy” or “digital citizenship” to “new media literacy” (Scolari et al., 2018; Xu et al., 2019). With the objective of contrasting social media threats, several countries have introduced educational initiatives to increase the awareness of students with respect to the detection of fake news and misleading information on the web.^2^ Still, due to their limited duration and their high costs compared to purely entertaining use of social media, the effects of these programs may be limited.

We propose a framework based on a virtual *Educational Social Media Companion* that enables continued, both in the classroom and outside, educational and interaction support for a community of learners, creating an *Educationally Managed Social Media Community* aimed at improving users' new media literacy and social media experience. Through companion support, the students can safely learn by doing how to deal with social media content, leveraging the positive aspects and counteracting the inherent threats. The relation between those elements is shown in Figure 1.

While previous educational attempts have focused on literacy activities mainly about *external* threats, improving the impact of social media on our society is challenging essentially because the interactions between users determine the quality and consequences of their experience. Rising awareness about the effects of own actions on the community members' experience and the importance of performing healthy interactions to realize a desirable condition notwithstanding the anonymity (Peddinti et al., 2014; Schlesinger et al., 2017) and deindividuation that social media may foster (Diener et al., 1980; Postmes and Spears, 1998; Lowry et al., 2016) is central in the presented educational endeavor.

We propose that the educationally managed communities participate in the description of a shared vision of a “desirable social media community” in terms of an operational Collective Well-Being (CWB) definition specific for their community (see Section 4). This will support the coherent formulation of community regulations, objectives and educational activities that involve several ethical issues entailing the definition of boundaries and trade-offs to own personal behavior online (see Section 4.1.1), such as enabling collective satisfaction and preserving the right to free speech (Webb et al., 2016) while facing the conflicts generated by users' different attitudes, opinions, personal history, and conflicting interests. A formalization of the CWB informs the CWB-RS, the companion recommender system aimed at recommending educational activities and content while balancing the recommendation incoming from the external social media platforms to improve the community's collective well-being, see Section 5.

### 3.1. An educationally managed social media community

The Companion safeguards teens' interactions on social media and implements *playful adaptive educational strategies* to engage and scaffold them considering personalized *educational needs and objectives*. These strategies comprise *scripted learning designs* (Amarasinghe et al., 2019) that informing by the CWB-RS will articulate the behavior of the Companion presenting teens with the right level of educational scaffolding (Beed et al., 1991) through an adaptive, personalized and contextualized sequence of *learning activities* and supported social media interaction—incorporating behavioral and cognitive interventions (*nudges* and *boosts*) that are grounded in behavioral psychology (Thaler and Sunstein, 2009; Hertwig and Grüne-Yanoff, 2017; Purohit et al., 2020). Game mechanics based on a *counter-narrative* (Davies et al., 2016) approach will support learning activities related to rising awareness: motivation, perspective taking, external thinking, empathy, and responsibility. These narrative scripts pursue collective and individual *engagement* with the Companion, offering motivating challenges and rewards aimed at keeping users' interest even in the presence of non-educational social media platforms (Van Staalduinen and de Freitas, 2011) while maintaining awareness of the digital addiction threat. The autonomous capabilities provided by the CWB-RS to the Companion can be particularly helpful outside of the classroom to avoid the cognitive overload, addiction or over-exposure to toxic content that the recommender system of an external, non-educational, social media platform may select. Moreover, they allow achieving a level of availability comparable with that of non-educational social media while reducing the moderating effort requested from the moderating educators.

#### 3.1.1. Educators and the companion: A human in the loop view

In our framework, the educators not only use the companion for delivering tailored educational activities in the classroom but, together with the experts, participate in the moderation and support of the community as well as in the definition of its CWB and related educational strategies, which drive the Companion by informing the CWB-RS. The educators oversee the CWB-RS behavior playing a key “human in the loop” role (Nunes et al., 2015; Zanzotto, 2019). This alleviates the complexities faced by the CWB-RS, such as noise in the estimation of content toxicity (see Section 5.4), which may also lead to misinterpreting users' needs and possibly exacerbating their condition. While the CWB-RS will have implicit moderating behaviors, e.g., reducing the presentation priority of users' confrontational interactions, the educators will have a central role in arbitrating users' disputes as well as solving the conflicts that may emerge between different components of an “under-construction” CWB measure, such as between emotional health (Roy et al., 2018) of one user and freedom of speech of another.

#### 3.1.2. Adopting behavioral economics to support collective well-being

This educational effort aims to help users of social media make the right decision and teach them the necessary skills to get to that point. Strategies developed in the context of behavioral and cognitive sciences offer a well-founded framework to address this issue. In particular, we consider nudging (Thaler and Sunstein, 2009) and boosting (Hertwig and Grüne-Yanoff, 2017) to be two paradigms that have both been developed to minimize risk and harm—and doing this in a way that makes use of behavioral patterns and is as unintrusive as possible.

**Figure 2:**
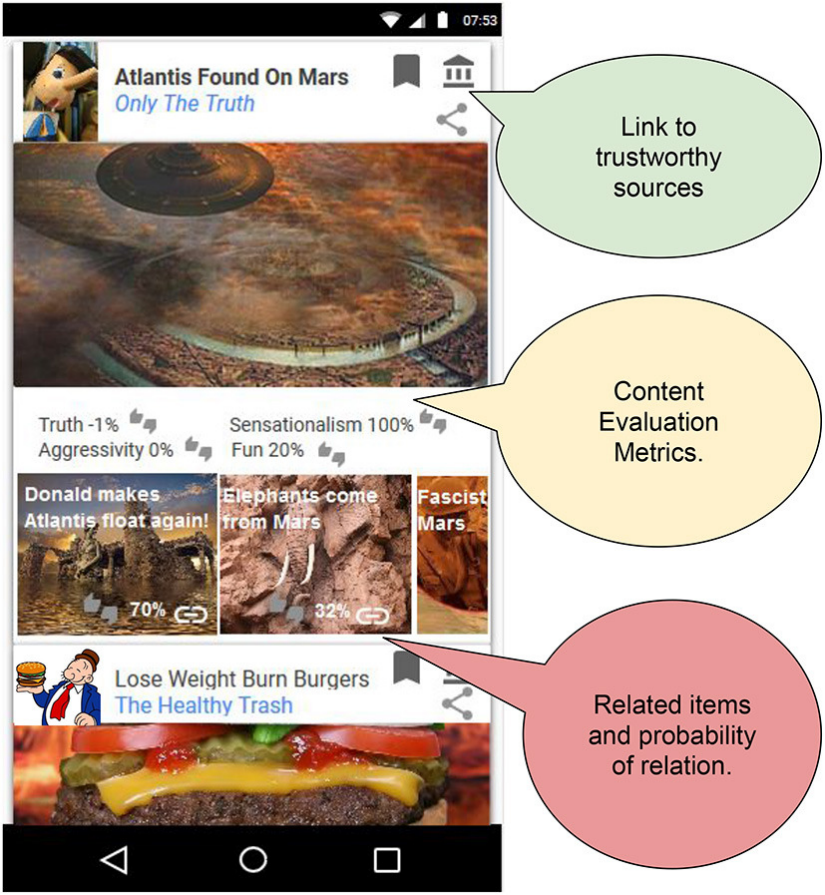
*Sketch of Companion User Interface*. The companion will support the students' interaction with social media by contextualizing the content to increase the students' awareness and allow them to access a more diverse set of perspectives (Bozdag and van den Hoven, 2015) and sources. It also explicitly and visually provides the students with an evaluation of the content's harmfulness (Fuhr et al., 2018). The example shows how a piece of imaginary fake news would be contextualized.

Nudging (Thaler and Sunstein, 2009) is a behavioral-public-policy approach aiming to push people toward more beneficial decisions through the “choice architecture” of people's environment (e.g., default settings). In the Companion context, such beneficial decisions could be to explore a broad range of different opinions about a specific topic and check understandable but scientifically correct pieces of information. In this working example, nudges could be implemented through a visual layout of the feed that allows easy exploration of such information (see Figure 2). Other forms of nudging are warning lights and information nutrition labels as they offer the potential to reduce harm and risks in web searches, e.g., Zimmerman et al. (2020).

The limitation of nudges is that they do not typically teach any competencies, i.e., when a nudge is removed, the user will behave as before (and not have learned anything). This is where boosts come in as an alternative approach. Boosts focus on interventions as an approach to improve people's competence in making their own choices (Hertwig and Grüne-Yanoff, 2017). In the Companion context, specific educational activities have been designed aimed at teaching people skills that help them make healthy decisions, e.g., select/read/trust articles from authoritative resources rather than those reflecting (possibly extreme) individual opinions (see Section 7.1).

The critical difference between a boosting and nudging approach is that boosting assumes that people are not merely “irrational” and therefore need to be nudged toward better decisions. However, such new competencies can be acquired without too much time and effort and may be hindered by the presence of stress and other sources of reduced cognitive resources. Both approaches nicely fit into the overall approach proposed here. Nudges offer a way to push content to users, making them notice. Boosting is a particularly promising paradigm to strengthen online users' competencies and counteract the challenges of the digital world. It also appears to be a good scenario for addressing misinformation and false information, among others. Both paradigms help us educate online users rather than imposing rules, restrictions, or suggestions on them. They have massive potential as general pathways to minimize and address harm in the modern online world (Kozyreva et al., 2020; Lorenz-Spreen et al., 2020).

#### 3.1.3. Educational activities

The Companion must also provide a satisfying and engaging experience by using *novel hand-defined educational games and activities* based on the interactive counter-narrative concept and educational games. SM's entertainment aspect is preserved during the navigation modulated in taking into account CWB, suggesting activities, content, and contacts for the user but managing the exposure to potential threats and addiction.

The Narrative Scripts help raise users' awareness about SM threats and train the students against them. They are sequences of adaptive learning tasks that provide the right level of educational scaffolding to individuals in developing critical thinking skills, including awareness—perspective taking, motivation, external thinking, empathy, and responsibility) by interacting with narratives, counter-narratives, and peers. These tasks can be different activities, including free-roaming inside the platform, guided roaming following a narrative, quizzes, playing minigames, or participating in group tasks. Different counter-narratives can be triggered depending on students' detected behavior (Lobo et al., 2022).

Counter-narrative are used to challenge biased content and discrimination, highlight toxic aspects of messages and attitudes, challenge their assumptions, uncover limits and fallacies, and dismantle associated conspiracy and pseudo-science theories.

Through a game-oriented setup, the companion bridges the “us” vs. “them” gap that is fostered by hate speech and other expressions of bias (e.g., gendered) and brings forward the positive aspects of an open society and focuses more on “what we are for” and less on “what we are against.” The users will be informed and requested to actively and socially contribute to creating and sharing content and material that fosters and supports the idea of an open, unbiased and tolerant society. Thus, the games can also offer the chance to build connections between the users, which, when isolated, are more vulnerable to online toxic content. One approach is to propose periodically specific tests and activities related to each threat, such as Szymanski et al. (2011).

A use case scenario is presented in Section 6 and the outcomes of several pilot studies that lie the basis for the educational activities are presented in Section 7.1.

#### 3.1.4. External and internal SM communities separation allows for educational opportunities

The Companion's location allows it to act as an interface between the educationally managed social media community and the external one. It permits mitigating the effect of external toxic content and offers the opportunity to recreate different interesting experiments about SM phenomena, such as the ones presented in Bail et al. (2018) and Stewart et al. (2019). A controlled environment in which social network dynamics are emulated can be adopted to stimulate students to understand SM mechanisms better, e.g., see Lomonaco et al. (2022a). Nowadays, the interactions intervening in social media are often mediated by automatic algorithms. Most teenagers ignore these dynamics that heavily influence their content and behavior when virtually interacting (Kuss et al., 2013). For example, in a classroom, it may expose sub-groups to recommendations with different biases or allow the students to change the recommender parameters (Bhargava et al., 2019; Lomonaco et al., 2022a).

#### 3.1.5. Companion exposes social media threats

The Companion's autonomous mechanisms will support the students in interacting with the social media content both inside (as a support learning activities) and outside (students' daily social network use) of the classroom. The Companion interface exposes its filtering and recommendation algorithms by allowing direct control of their parameters (Bhargava et al., 2019). It will contextualize the content to increase the students' awareness and allow them to access a more diverse set of perspectives (Bozdag and van den Hoven, 2015) and sources (see Figure 2). It also explicitly and visually will provide the students with an evaluation of the content's harmfulness (Fuhr et al., 2018) (see Section 5.4).

## 4. Defining a collective well-being metric for social media

Social media is an integral part of our everyday lives that is having both negative and positive effects (Wang et al., 2014; Chen et al., 2017). Hence, as positive aspects rely on the same mechanisms exploited by threats, and because each user's behavior will affect the other members of the community while values can differ between communities, it is desirable and necessary to explicitly and collaboratively define shared community principles corresponding to the desired condition of the community. These community principles will constitute the foundation to define a specific measure of the overall impact of social media in the community at an individual and a societal level, that is, to measure the desirability or Collective Well-Being (CWB) of a certain condition of the social media community (Roy et al., 2018). These community principles, formalized in the CWB measure, together with an understanding of the virtual and physical social dynamics in the community, should drive the definition of users' behavior guidelines and connected educational objectives to reach and maintain the community in the desired condition, or in other words, to achieve a high level of CWB. A quantitative measure of CWB allows for a more accurate evaluation of the impact of different aspects of the interaction on the community while taking into account the complex and fast dynamics of social media. When CWB is estimated directly on the SM platform it could allow directing its autonomous components, e.g., recommenders, to collaborate in achieving the desired community condition. This would be a more democratic and transparent objective than the ones currently pursued by the social media platforms (Gorwa, 2019). In our framework, it is used to direct the algorithms at the interface between the educationally managed community and the external social media.

### 4.1. Research on collective well-being and social media

The literature presents several definitions and measures of well-being (Topp et al., 2015; Gerson, 2018). Some of them were applied in the context of social media to estimate their effects (Mitchell et al., 2011; Kross et al., 2013; Wang et al., 2014; Chen et al., 2017; Verduyn et al., 2017) but mostly considering the single individual with limited consideration for the overarching social aspects (Helliwell, 2003).

Gross Domestic Product (GDP) has been proposed as an index of the economic well-being of a community.^3^ In such contexts, inequality is also an important factor, and it is common practice to use the Gini index to measure it (Osberg, 2017). While the economics view is difficult to connect to a social media context, they share similar key issues: which aspects to measure and, above of all, how to compare and aggregate measures of individuals' well-being to synthesize that of the whole society (Costanza et al., 2014), even if in this work we consider only the local educational community.

Multidisciplinary notions of CWB extend that of individual well-being to measure a group-level property (construct). They include community members' individual well-being incorporating diverse domains, such as physical and mental health, often stressing the presence of positive conditions. They study which properties of the community affect the members and how much each of these properties adds to a comprehensive measure of collective well-being. We already stressed the importance of education and educational objectives to support constructive interactions and achieve desirable community conditions, i.e., a high level of well-being. However, education itself is often already part of well-being frameworks (White, 2007; Michalos, 2017; Spratt, 2017b; Roy et al., 2018). The connection between education and well-being has been analyzed from several perspectives. In our framework, the most relevant one is the one defined as *social and emotional literacy* in Spratt (2017a).

Roy et al. (2018) present a CWB framework divided into different domains and comprising health-care and non-health-care-related community factors where the contribution of the latter ones is supported by evidence of their effects on health. This framework can help to define a checklist for the definition of a community-specific CWB and related measures and indicators. We show in Table 1 the properties that may be relevant for education and social media communities for the following reasons:

- *Opportunity* domain is related to “the perceived opportunity to achieve life goals and socioeconomic mobility” (Diener and Seligman, 2006) as well as the access to education. Social media can be a powerful tool for accessing many opportunities. Feeling in control while using them, instead of just a distraction or worse an addiction, may be an important part of CWB for SM;
- *Connectedness* domain is related to the presence of supportive, high-quality, reciprocal relationships with secure attachments. Includes dimensions of social acceptance and social integration that depend on the behavior of other members of the community (Van Der Maesen and Walker, 2011);
- *Vitality* domain covers many emotional aspects of several individual well-being definitions, such as Fredrickson's one and Seligman's model of flourishing (Fredrickson, 2004; Seligman, 2012). However, spillover effects (Helliwell, 2003) and emotional influence make vitality an important aspect also at a social level;
- The threats presented in Section 2 would impact negatively the affects component of the *Vitality* and *Connectedness* domains;
- The *Contribution* domain relates to community engagement and related feelings of meaning and purpose. Contribution can improve other members' experience but may also have negative effects;
- The *Inspiration* domain relates to creativity and lifelong learning, areas where social media have a huge potential.
- The psychosocial *Community* characteristic that is clearly relevant for social media settings:

“*A community with a negative psychosocial environment is one that is segregated and has high levels of perceived discrimination and crime, high levels of social isolation and low community engagement, and low levels of trust in government and fellow citizens.”* (Mair et al., 2010; Klein, 2013; Engel et al., 2016).

**Table 1:** Categories of properties of social media communities relevant for collective well-being and education extracted from the framework presented in Roy et al. (2018).

**Categories**	**Description**	**References**
Vitality	“The vitality domain includes... emotional health, with positive and negative affect, optimism and emotional intelligence.”	Hong et al., 2017
Opportunity	the “perceived opportunity to achieve life goals and socioeconomic mobility,” “influenced by ... access to education and training”	
Connectedness	“The connectedness domain assesses the level of connection and support among community members... Human relationships and relatedness are fundamental for the achievement of well-being according to many foundational theories of well-being.…Connectedness includes dimensions of social acceptance (i.e., positive attitudes toward people) and social integration (i.e., feeling a sense of belonging to the community).”	Dunn, 1959; Cohen and Wills, 1985; Fredrickson, 2004; Ryff et al., 2004; Lopez and Snyder, 2009; Seligman, 2011; Van Der Maesen and Walker, 2011
Contribution	“The contribution domain incorporates residents' feelings of meaning and purpose attributed to community engagement and belonging (e.g., volunteering, civic engagement, or belonging to a religious or community group). Sense of purpose is a cognitive process that provides personal meaning and defines life goals.”	Forgeard et al., 2011; Keyes, 2012; Roy et al., 2018
Inspiration	“The inspiration domain includes community members' perceived access to activities that are intrinsically motivating and stimulating… [such as] life-long learning, goal-striving, creativity, and intrinsic motivation.”	Meier and Schäfer, 2018; Roy et al., 2018

Community is partially overlapping with the Connectedness and Contribution domains but describes aspects that are easier to concretely measure in social media networks.

While these formulations of CWB can inspire a guideline to define social media communities' principles and CWB metrics, they must be extended and formalized to better take into account the specific issues and opportunities of SM and in particular, the threats reported in Table 2. Another important aspect to address is combining contrasting factors or, in other words, formalizing the complex ethical decisions induced by the conflicts and trade-offs that emerge in any social context (Müller, 2020).

**Table 2:** Examples of social media threats distinguished into three categories (content, algorithmic, network, attacks, and dynamics) and examples of cognitive phenomena that may exasperate them.

**Content based social media threats**	**Social media cognitive and socioemotional threats**
Toxic content (Kozyreva et al., 2020)	Impulsivity (Lee et al., 2019)
Fake news/disinformation (de Cock Buning, 2018)	Fear of Missing Out (Alutaybi et al., 2019)
Bullying (Grigg, 2010; Mladenović et al., 2021)	Confirmation bias (Knobloch-Westerwick and Kleinman, 2012; Del Vicario et al., 2017)
Hate speech (Zimmerman et al., 2018)	Social reinforcement (Liu et al., 2018)
Stalking (Tartari, 2015)	Backfire effect (Bail et al., 2018)
Discrimination (Stoica et al., 2018)	Attention limit (Weng et al., 2012)
Radicalization (Johnson et al., 2016)	Emotional load (Kramer et al., 2014; Brady et al., 2017)
Smoke (Christakis and Fowler, 2008)	Anonymity (Urena et al., 2019)
Sexism/sexual harassment (Barak, 2005)	Depersonalization (Diener et al., 1980; Postmes and Spears, 1998)
Objectification (Ozimek et al., 2017)	Digital addiction (Kuss and Griffiths, 2011; Brand et al., 2014; Almourad et al., 2020)
Beauty stereotypes (Verrastro et al., 2020)	Lack of digital literacy (Whittaker and Kowalski, 2015; Xu et al., 2019)
**Social media dynamics induced threats**	**Algorithmic social media threats**
Filter bubbles (Bozdag and van den Hoven, 2015; Nikolov et al., 2015; Geschke et al., 2019)	Content diversity (Adomavicius et al., 2013)
Echo chambers (Gillani et al., 2018)	Misclassification (Stöcker and Preuss, 2020)
Digital wildfire Webb et al. (2016)	Algorithmic bias (Chen et al., 2020)
	Malicious users (Zhou Y. et al., 2017)
	Gerrymandering (Stewart et al., 2019)

#### 4.1.1. Challenges of defining collective well-being for social media

Defining a CWB metric for SM is an ambitious endeavor that requires a combined effort of different disciplines. It would range from political sciences, sociology and psychology over ethical considerations all the way to computer science, machine learning and network theory. Besides CWB aspects for physical societies, the impact of integrated intelligent agents must also be taken into account in the context of social media, as discussed in Sections 2.2, 2.3. A CWB measure for virtual communities has to take into account the conflicts between members as they are frequent and algorithmically augmented. Therefore, the conflict between the right to freedom of expression, user satisfaction, and social impact must be stressed more when defining a social media CWB than with physical societies where these factors have slower and better-understood effects and may have regulations already in place (Webb et al., 2016).

Conflicts between members' interests pose serious ethical concerns that are out of the scope of this paper and have been the focus of recent research in AI and ethics in different domains (Cath et al., 2018; King et al., 2020; Milano et al., 2021). When social media are integrated into an educational framework, the problem may be mitigated by involving educators and experts as moderators. We propose that such an educational setup can also allow initial studies of the implications of a social media platform that aims to improve CWB.

### 4.2. Participative definition of social media community principles and CWB factors

Social media community principles and corresponding CWB factors must be shared by the members of the community. While research in the field can inform about common social aspects, internationally acknowledged human rights, or social media-specific phenomena, a community would most likely have the freedom to define tailored principles. To achieve this human-centered approaches to the participatory design of technology are being explored by the researchers. These approaches involve the stakeholders in the analysis of relevant factors and the co-design of technological solutions. One of the main challenges is bridging the gap between the community members' knowledge and the complexity of cyber-social systems like social media (DeVito et al., 2018). An example is a qualitative study to explore adolescents' representations of social media based on pictorial metaphors, reported in Sánchez-Reina et al. (2022). The study proposed and analyzed the outcomes of a school project entitled “The Social Media of the future.” Discourses and visual representations of a total of 168 drawings about their visions for their ideal Social Media tools were analyzed. The results of the analysis pointed out that the relevant CWB factors shared by the adolescents participating in the study were: care about additive features, transparency in the conflict of interest behind the SM business, also in terms of agency to be able to monitor and control privacy and security facets.

### 4.3. Toward the automatic estimation of collective well-being in social media communities

Social media are strongly integrated with information systems that can affordably offer a huge amount of data with a high frequency. Transforming this data for the estimation of suitable collective well-being measures through machine learning methodologies would open the way to many research and applicative opportunities, such as autonomous systems that maximize CWB and avoid current issues induced by profit-based objectives.

Current CWB formulations are not easy to estimate directly using data available in real-time on social media, which is necessary to support an autonomous system optimizing CWB. Moreover, such formulations need to be extended to take into account specific social media issues. For example, most of the available formulations of collective well-being focus on positive aspects. Nevertheless, the positive aspects (see Section 4.1) and negative ones (see Section 2) need to be explicitly considered as part of the CWB as they strongly affect social media users and in particular teenagers.

We propose to define a *collective well-being metric for social media* by combining suitable components of classical CWB and SM threat measures. The measures of these components could be measured by periodically proposing specific surveys and activities (Loughnan et al., 2013). However, we propose that additional richer and more transparent measurements can be performed by developing intelligent components that analyze users' behaviors. In this definition, for each user, event, i.e., content or connection related, and aspect defined relevant for the CWB three terms are computed:

- **CS(aspect, user)** Content Shared measures the aspect-specific value of the content shared by the user;
- **CE(aspect, user)** Content Exposure measures the aspect-specific value of the content observed by the user;
- **CC(aspect, user a, user b)** Contact Creation measures the aspect-specific value of new connections based on the participants' CS and CE.

These elements account for the double role of each member of the social media community as both receivers and producers of content. In our educational setup, where only the community of interest is in contact with an external social media community, we distinguish between “endogenous” and “exogenous” aspects. The community can be exposed to threats that are generated outside but a community can also generate such threats inside as part of the interactions in the social medium. In this case, the feeds from external sources may be weighted differently.

While the CS can be seen as a direct expression of the state of the user, it strongly depends on the user's style of interaction. Moreover, only relying on the content shared by users would induce a substantial delay compared to the moment when a user got actually affected by observing a piece of content (CE). Conversely, the user is exposed to a multitude of diverse inputs hindering the interpretation of the overall effect only from the CE, while the user's reactions (CS) may be more indicative of the most impacting events. Indeed, current affective state estimators and toxic/positive content detectors can only provide noisy estimations of the current user state and the content quality. However, the availability of complementary data with higher reliability is limited.

Once each event is scored for each aspect of interest, it must be decided how to aggregate these terms over users, time, and the different aspects to obtain an estimation of the total CWB of the community. Indeed, the definition of an actual metric following this strategy requires making a number of choices. For example, about the scale for the terms of different aspects considered. Regarding aggregation over time, CC, CE, and CS values could be simply averaged. Other approaches could be considered to take into account the frequency of the events or the diversity of opinions presented or give more relevance to extreme events, which may be more accurately detected and evaluated. In particular, the value of being exposed to multiple opinions (time-aggregated CE) may be augmented with a measure of diversity (e.g., entropy) (Garimella et al., 2017; Matakos et al., 2022).

Clearly, the design of the CWB metric presents a number of challenges requiring careful consideration even for small educational communities that our framework targets. In devising their solutions often the naive approach may at best be ineffective, and at worst exacerbate the issues it was intended to solve. For example, the aggregation over the aspects dimension may not seem complex when considering the aspects to be independent. In reality, the impact of the various aspects on the users may be interlinked, for example over exposure to content focused on one aspect (e.g., videogames) may lead to overuse of the platform or tire the user who will lose the opportunity to learn about more important content (e.g., social issues).

The most complex aggregation to design is over users because it has to balance the well-being of different individuals and groups of users taking into account their conflicting interactions along different dimensions. It is important to consider the different features of each user while respecting privacy constraints. For example, vulnerable users are often victims of toxic content but also producers (Bessi, 2016; Bronstein et al., 2019; March and Springer, 2019), which affects the CS value. It is important that they are not isolated (Burrow and Rainone, 2017) and that, at the same time, the toxic content should not be fed to those who could be more affected and instead presented to educators or other community members that have shown constructive reactions to such type of content. This means that the content exposure (CE) should be differently weighted for different community members based on their resilience and that supportive connection creation (CC) should be favored between people with high and lower resilience. Still, it is important that resilient members are not overloaded with toxic content and support responsibilities (Steiger et al., 2021).

Apart from the weighting issue issues, another important format issue is the selection of the actual aggregation function across users. Adopting the naive average a society where a few radicalized users share extremely hateful content may have a higher CWB score than one with a number of users sharing content about action movies with slightly violent scenes. Another reason why a linear combination of components may not be suitable in the definition of a well-being measure is that it will simply induce maximizing the terms with positive weights and minimizing terms with negative ones, without allowing a balance. For example, if interactions between drastically opposite opinions are considered negative because of possible backfire effects and flames (Bail et al., 2018), and interactions between excessively similar opinions are also considered negative because of the echo chambers they may give place, then also interactions between moderately different opinions will have a negative value even when they may lead to a reduced polarization. Other aggregation functions may be chosen but it is still difficult to find general solutions. For example, defining the well-being of society as the well-being of the member with lower well-being (i.e., minimum instead of an average) could lead to focusing all the resources on factors that may not be actually changed.

### 4.4. Network measures for collective well-being on social media

Network-specific measures (Rayfield et al., 2011) can be an important part of an actionable CWB measure for social media. Several threats and well-being-related phenomena are implicitly defined in terms of network measures. These measures may also be particularly useful as proxies of future critical conditions without having to execute expensive simulations. For example, Moore et al. (2021) show that the increase of a network measure of inclusiveness promotes the efficiency and robustness of a society. Stewart et al. (2019) show that an unbalanced network structure may lead to suboptimal collective decisions. Effects of positive and negative interactions at a network level have been studied in Leskovec et al. (2010). Concepts like social influence and homophily (Aral et al., 2009; Guo et al., 2015) play an important role in the formation of different network conditions, like segregation, that are crucial for CWB. The diversity measures already proposed as part of the CE, CS, and CC elements would also contribute to a higher CWB rating for diversified and integrated communities than polarized and segregated ones. Other measures viable to characterize user roles, such as centrality and closeness, can also be used to aggregate the individual users' threat scores over the network (Drachsler et al., 2009; Manouselis et al., 2011).

## 5. An educational collective well-being recommender system

Recommendation systems (RSs) are ubiquitous in online activities and are crucial for interacting with the endless sea of information that the Internet and social media present today. In social media platforms, they have introduced the possibility of personalizing suggestions of both content and connections based on the use of user profiles containing also social features (Heimbach et al., 2015; Chen et al., 2018; Eirinaki et al., 2018). Their goal has been to maximize the users' engagement in activities that support the platform itself. However, these self-referential objectives fail to consider repercussions on users and society, such as digital addiction (Almourad et al., 2020), filter bubbles (Bozdag and van den Hoven, 2015), disinformation wildfire (Webb et al., 2016), polarization (Rastegarpanah et al., 2019), fairness (Abdollahpouri and Burke, 2019; Ranjbar Kermany et al., 2021), and other issues discussed in Section 2. To address this, we propose the concept of *Collective Well-Being aware Recommender Systems (CWB-RS)*. The CWB-RS extends social media RS intending to maximize the cumulative long-term *CWB metric* instead of self-referential platform objectives. Compared to previous efforts in dealing with possible negative effects of RSs (Abdollahpouri and Burke, 2019; Rastegarpanah et al., 2019; Ranjbar Kermany et al., 2021), the CWBRS takes into account multiple issues and, to reduce their cumulative impact on society, it adopts longer terms strategies fitting into our educational framework.

Integrating educational objectives aimed at achieving *CWB* in the longer term the CWB-RS will also have functions similar to those of a (collective) *Intelligent Tutoring System* (Greer and Mark, 2016). RSs have been widely used in educational settings (Manouselis et al., 2011), and they are receiving increasing attention due also to the fast growth of MOOC (Romero and Ventura, 2017) and the availability of big data in education (Seufert et al., 2019). In educational contexts, recommendations are sequential and functional to achieving learning goals (Tarus et al., 2017). Similarly to the social media context, they have also employed social information (Kopeinik et al., 2017; Elghomary and Bouzidi, 2019). However, they are usually acting on the content provided by educators with educational aims, while CWB-RS also has to redirect disparate content flowing from external Social Media toward achieving educational objectives.

**Figure 3:**
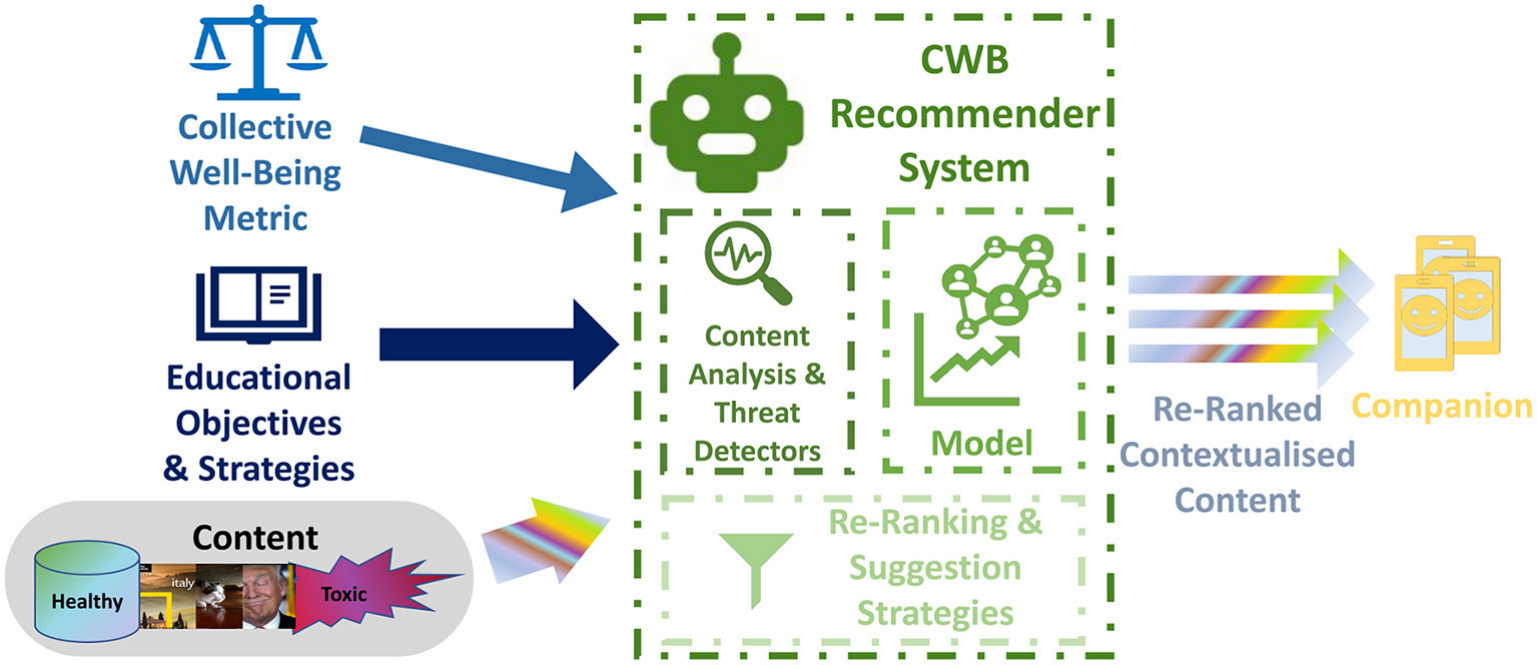
*Role of the CWB-RS in the Companion*. CWB-RS will process the *content generated by the users* of the *educationally managed social media* and the *content externally recommended* for them by the RSs of the external social media platform to create new recommendations aimed at maximizing the cumulative long-term *collective well-being metric*. *Content Analyzers and Threat Detectors* will analyze and evaluate the level of threat for each piece of content and other relevant information as the users' emotional state. This information will be used to: 1) *augment* the information provided to the users by the companion interface; 2) *evaluate* through *predictive models of users' opinions and reactions* the future effects of different sequences of re-ranking and recommending actions; 3) *select* the re-ranking and recommending actions that resulted in the highest expected cumulative improvement in terms of learning objectives, CWB metrics, agreement with selected educational strategies and user engagement.

As shown in Figure 1, the CWB-RS creates new recommendations presented through the Companion by processing both the content generated *internally* by the members of the *educationally managed social media* community and the content recommended for them by the RSs of the *external* platform. *Content Analyzers and Threat Detectors* (see Figure 3 and Section 5.4) will analyze each piece of content to evaluate the level of threat and other relevant information for the CWB metric, such as the users' opinions and emotions (see Section 4.3). This information will be used to: 1) evaluate the current condition of the users; 2) *augment and contextualize* the content provided to the users; 3) *evaluate* the future effects of different sequences of content re-rankings and recommendations through predictive models of users' conditions; 4) *select* the actions that account for the highest expected, long-term, cumulative CWB metric.

### 5.1. Educational directions for the CWB-RS

CWB-RS educational objectives are designed by educators and experts (see Section 3). They can be encoded in terms of measures related to specific threats or other well-being variables, such as those extracted by *content analyzers and threat detectors* allowing to easily combine educational and regular CWB objectives (Van Seijen et al., 2017). Different approaches have been proposed to effectively combine and scale multiple terms in objective functions (Harutyunyan et al., 2015; Marom and Rosman, 2018). These objectives express how much each student: (a) is conscious of his role in other users' well-being, (b) improves his behavior, and (c) is having a healthy experience. For example, an objective would be “curb obsessive selfies posting” (Ridgway and Clayton, 2016), which would act on the content shared (CS) for the aspect “selfies.” Another example could be breaking the filter bubbles focused on racist content and helping users hold an unbiased mindset (reduce both CE and CS on the aspect “racism”). In this case, the connected recommendation strategy will be to provide content with opposite but not confrontational perspectives (Bozdag and van den Hoven, 2015; Garimella et al., 2017; Matakos et al., 2022). This strategy can be combined with educational games proposing specifically themed challenges, such as finding pictures of achievements performed by people of different ethnicities, suggesting changing the recommender filter parameters directly, or just reducing the racist content presented and substituting it with low harm feeds. The CWB-RS can also recommend content to asses the current student's condition (Zhou et al., 2010; Kunaver and Požrl, 2017) to inform successive personalized interaction.

Educators and experts will also define interaction strategies specific to each objective (Griffith et al., 2013). Sketches of *high-level CWB-RS educational strategies* will be hand defined by the educators and experts to choose between the different educational objectives for each student in an effective and contextualized manner. *Lower-level educational strategies* for the CWB-RS comprise hand-defined *learning activities* and *minigames* as well as modulation of the recommendations, for example, showing diverse content as tests to explore students' preferences.

Engagement is an important factor for both social media platforms (Wu et al., 2017; Zheng et al., 2018) and educational activities (Sawyer et al., 2017). The CWB-RS must prevent students from “dropping out” (Eagle and Barnes, 2014; Yukselturk et al., 2014) and moving to non-educational social media. In a complementary manner to the game-oriented motivational mechanisms of the Companion (Van Staalduinen and de Freitas, 2011), the CWB-RS must therefore preserve a healthy level of engagement (Arroyo et al., 2007; Chaouachi and Frasson, 2012; Mostafavi and Barnes, 2017; Zou et al., 2019) while avoiding excessive exposure to toxic content as well as any form of addictive use (Lavenia, 2012; Nakayama and Higuchi, 2015; Almourad et al., 2020).

### 5.2. Challenges in social media RS and CWB-RS

The realization of effective social media recommendation systems, as reviewed in Chen et al. (2018) and Eirinaki et al. (2018), presents several challenges that in recent years have brought drastic changes to the field. In particular, some of the biggest challenges are the highly diverse information they process (e.g., content, trust, connections), the complex dynamics of the interactions, the fast pace of growth of the social graph, and the enormous amount of multimedia and textual elements to process (Covington et al., 2016; Eksombatchai et al., 2018). In the case of the CWB-RS, the size of the internal social network is limited (i.e., the number of students) and a big part of the data will come preselected by the external RS, thus forming an implicit two stages approach (Borisyuk et al., 2016; Covington et al., 2016; Ma et al., 2020) with only the second stage in charge of the CWB-RS. However, the creation of a CWB-RS presents several other theoretical, technical and ethical challenges that are mostly not faced by classical RS.

#### 5.2.1. Diverse internal and external content

A first demand for the CWB-RS is to combine content defined by the members of the educationally managed social media with recommendations from the external social media. While this controlled separation from the external platforms offers the opportunity for novel educational experiences, the heterogeneous nature of signals and structures poses the question of how to combine them. This is all conceptually similar to some of the major challenges and opportunities of enterprise and intranet search compared to general web search (Hawking, 2010; Kruschwitz and Hull, 2017).

#### 5.2.2. Social information

In classical social media RSs, the use of social information is relatively straightforward. For example, connections between users can be interpreted as a cue of similarity between their interests. For a CWB-RS, sharing content based on social connections may spread toxic content, however, it can be useful if one of the connected users has exemplary behavior. Moreover, social network structures affect not only information propagation but also decision and behavior (Stewart et al., 2019). Thus in CWB-RS, some properties of the structure of the social connection graph of the internal community may be part of the objective (e.g., CC in Section 4.3). Still, the recommendation and creation of connections between diverse groups may sometimes lead to toxic behaviors, e.g., backfiring (Bail et al., 2018).

#### 5.2.3. Lack of direct reference information for the CWB-RS

Classical RSs maximize the users' satisfaction and engagement, usually estimated through accessible proxy measures, such as time of usage or likes. These allow the definition of reference information or teaching signals to improve the RSs behavior based on the similarity between items or between users' previous selections (Wu et al., 2017; Eirinaki et al., 2018). These signals do not inform about the level of CWB or achievement of user-specific educational objectives. The CWB-RS needs both to estimate less accessible quantities, such as knowledge acquired or behavioral improvement, and to recommend content taking into account the users' learning trajectories, comprising their current state and assigned objectives. Still, these measures do not easily translate into future recommendations. For example, if a recommendation led a student to achieve an educational goal, this does not imply that it would be useful to suggest similar content to the same student again, as it will not provide him with new educational information. It may still indicate that it is useful to suggest similar content to other students who have to achieve the same goal.

#### 5.2.4. Temporal aspects and sequence of recommendations

Classical RSs regard recommending as a static process mainly focusing on “the immediate feedback and do not consider long term reward” (Liu et al., 2018; Zhao et al., 2019a). Instead, to achieve lasting CWB and the related educational processes, it is necessary to account for the effects of sequences of recommendations. For example, sequencing of lectures, tests, and feedback, is common in most educational strategies. In addition, a classical RS does not consider the interdependence between users' preferences and the RS recommendations, which is crucial to model and counter the filter bubble and echo chamber phenomena. Another reason for the CWB-RS to consider a temporal dimension is to enable the use of an accurate dynamic model of the students and the natural variation of their preferences (Zeng et al., 2016). This allows, for example, to prepare the conditions and select the best time for exposure to content aimed at improving students' empathy as well as avoiding wrong conditions, such as those with a high level of user stress, when such content would be ignored or even lead to backfire (Bail et al., 2018).

### 5.3. CWB-RS adaptation and personalization through Reinforcement Learning

The Reinforcement Learning (RL) paradigm adoption to drive the adaptation and personalization of the CWB-RS behavior (Zhao et al., 2019a; Zou et al., 2019) is a natural solution to the sequential control, lack of supervised teaching signal, and the other technical issues described above. RL-based recommender systems are recently gaining attention in the community (Shani et al., 2005; Liu et al., 2018; Zheng et al., 2018) because of their flexibility, and the growth of the deep reinforcement learning field (Mnih et al., 2015; Zheng et al., 2018). As suggested in Zhao et al. (2019a), RL-based RSs allow solving not only the problem of frequent updates of the user profile, typical of RS in social media, and offer also a precise formulation of the initialization problem in terms of exploitation-exploration (Iglesias et al., 2009; Hron et al., 2020).

From a machine learning perspective, *CWB-RS* educational objectives, learning strategies and activities, can be respectively seen as manually defined rewards, sub-goals, and sub-policies in a Hierarchical Reinforcement Learning (HRL) framework (Zhou et al., 2019, 2020) which improves its adaptation performance by breaking down the high-level decisions (e.g., the educational objective a student must achieve) and the step-by-step decisions (e.g., which activity or content to show at the moment). This reduces the computational costs and amount of data necessary to derive the educational policy and objectives directly from the long-term optimization of the CWB metric (Barto and Mahadevan, 2003).

Both classical RL (Iglesias et al., 2009; Dorça et al., 2013; Zhou G. et al., 2017) and HRL have been used in ITS (Zhou et al., 2019, 2020) and RS. To our knowledge, this is the first time they are combined. While the field of RL-based ITS is still young and presents several limits (Zawacki-Richter et al., 2019), it could address the complex problem of supporting students dealing with the diverse and enormous environment of social media. Still, the additional flexibility of RL-based RS comes at the cost of higher complexity, particularly in terms of training and evaluation setup (Henderson et al., 2018), as well as deploying in real-world applications (Dulac-Arnold et al., 2019; Rotman et al., 2020).

#### 5.3.1. Difficulty of creating CWB-RS datasets

Reinforcement Learning systems developed to act in real-world conditions are usually pretrained offline on available datasets. Much of the solution quality depends on the similarity between the dataset and the application setting (Rotman et al., 2020). The creation of real-world reinforcement learning datasets most often requires *ad-hoc* solutions.

The collection of CWB-RS datasets must take into account the users' profiles, which may be gathered using a self-reported survey, as in Khwaja et al. (2019), as well as users' neighborhood information, behaviors (e.g., posts) and observations (e.g., recommendations). Mining this information, however, needs to comply with privacy and company policies. Additional challenges are presented by the necessity to cover the various reactions that students may have under exposition to combinations of disparate social media (Zhao et al., 2019a). Social media show a complex interplay between the individual, social, and technological levels of filtering (Gillani et al., 2018; Geschke et al., 2019), with substantial effects on users' behaviors. Therefore, one of the strongest challenges is washing out the effects of the RS adopted during the data collection, which functioning is usually unknown, enabling the use of the dataset to train a CWB-RS that could propose diverse recommendations and induce different selections.

*Crowdsourcing* (Boudreau and Lakhani, 2013) can be used for large-scale evaluations or for creating datasets under limited periods (Kittur et al., 2008). However, special care needs to be taken to ensure the reliability of crowd data (Buhrmester et al., 2018) as the seriousness with which volunteers take their interactions with the system can be limited. These complexities demand to devise an effective strategy to build a real-world dataset that considers including the micro-, meso-, and macro-structure, different sources, and modalities.

*Model-Based RL* For the specific setting of the educationally managed social media community, the task is simplified considering the reduced content variety compared to the external community. Also, while a CWB-RS must be aware of the condition and behavior of the entire community, this may be factored in terms of the dynamic models of its members. Using different combinations of the same members' models, it could be possible to create different community models that allow a broader set of training conditions for the CWB-RS in simulation. They will also enable online simulations for estimating the results of a sequence of recommendations (see Figure 3 and Zhao et al., 2019b; Schrittwieser et al., 2020). The literature on interaction models for social media is extensive. Szabo and Huberman (2010) were one of the first to show the importance of cognitive and content factors. The models proposed in Guo et al. (2015); He et al. (2015) reason simultaneously on the patterns of propagation and the topics. Most of these models do not account for user adaptation, which is crucial in this context. However, the solution could be to adopt generative models of adaptive user behaviors, such as Das and Lavoie (2014), Lindström et al. (2019), and Ognibene et al. (2019). While these studies and many more led to improved forecasting systems, there is a consensus that there are intrinsic problems that limit the predictive power with both sufficient accuracy and anticipation, see for example Cheng et al. (2014). A significant improvement of baseline algorithms requires very detailed information about the community (Watts, 2011). However, the CWB-RS has access to rich information about the educationally managed network. This, together with its limited, size will improve the efficacy of the predictive models.

#### 5.3.2. Risks in the exploration phase of RS based on RL

Reinforcement learning can provide online adaptation to conditions that detach from the training set used for offline pretraining. However, this comes with exploration costs that in real environments can pose prohibitive risks (Rotman et al., 2020). Even if the CWB-RS is not facing critical safety tasks like those of self-driving systems, repeated sub-optimal recommendations may just reinforce the threats the Companion is trying to address. To alleviate these issues adaptive novelty detection methods (Rotman et al., 2020) will be implemented in the CWB-RS to recognize situations far from the agent experience and hand over the control to educators or a safe controller. Moreover, the HRL paradigm has been adopted for the CWB-RS to constrain and minimize exploration risks and costs (Nachum et al., 2018; Steccanella et al., 2020) while providing direct control and interpretability to the educators (Shu et al., 2017; Lyu et al., 2019). Ultimately, under the direction of learning objectives and strategies, the set of problems that the CWB-RS will have to solve would be limited to balancing reranking requests from different active strategies and prioritizing one objective over the few others defined in the current high-level learning strategy.

#### 5.3.3. Noisy rewards and action results

An additional constraint comes from the difficulty of characterizing the toxicity of the social media content (see Section 5.4) on which the RS must act. This results both in erroneous recommendations (e.g., content that was mistakenly supposed to be toxic undergoes reduced propagation speed) and stochastic rewards (toxic content is evaluated by error as healthy and a positive reward is provided to the CWB-RS from the CE and CS estimation). While the RL method accounts for noisy actions' results, they still affect the performance of the system, both in terms of execution and learning time. Regarding noisy rewards, literature has only recently started to provide solutions (Huang and Zhu, 2019; Wang et al., 2020). Still, it must be noted that in our setting, getting a positive reward for something that was considered positive should not crucially impair the acquired RS policy as the system allowed the propagation of something that it evaluated healthy (or toxic) and accordingly evaluated its reception by other SM users. Thus, in this case, the two errors may cancel each other out and take advantage of improvements in the detectors. Moreover, when applying RL for ITS, an additional strategy that can be leveraged to counter these issues is to use more reliable tests that would allow for evaluating the state of the users and provide more reliable rewards. Due to social media complexities, the effects of detectors' failures on the performance of CWB-RS can be heavy, with backfiring as the worse-case scenario. Extensive tests would be necessary both in simulation (e.g., Geschke et al., 2019 and real-life as well as comparisons with classical recommender systems for social media that are not sensitive to content toxicity.

### 5.4. Threat detectors and content analyzers

Social media threat detectors and content analyzers have multiple roles in the platform already described in Section 5. Given the importance of social media threats, as described in Section 2, researchers have been studying how to automatically identify them (some examples can be seen in Table 3). Several shared tasks have been proposed and each year they become more challenging. Moreover, new evaluation criteria, such as multilingual detection at Task 5 in Semeval 2019 (Basile et al., 2019), different domains at HaSpeeDe in Evalita 2020 (Hoffmann and Kruschwitz, 2020; Sanguinetti et al., 2020), detections at the spam level at Task 5 in Semeval-2021 (Pavlopoulos et al., 2021), and generalization to social media platforms other than those used in training at EXIST in IberLEF 2021 (Rodríguez-Sánchez et al., 2021), have been included in the datasets.

**Table 3:** Short list of works on social media threat detection and content analysis exemplifying the variety of approaches and works.

**Type of detector**	**References**
Stance detection	Augenstein et al., 2016; Zarrella and Marsh, 2016
Controversy identification	Hessel and Lee, 2019; Zhong et al., 2020
Fact-checking	Dale, 2017; Long, 2017; Wang, 2017; Jobanputra, 2019; Liu and Lapata, 2019; Nie et al., 2019; Atanasova et al., 2020
Hate speech	Cer et al., 2018; Basile et al., 2019; Indurthi et al., 2019; Nikolov and Radivchev, 2019
Violence recognition	Perronnin et al., 2010; Nievas et al., 2011; Bilinski and Bremond, 2016; Zhou et al., 2018
Gender bias	Prost et al., 2019
Offensive content	Hosseini et al., 2017; Zampieri et al., 2019

Those detectors are usually defined as a classification task commonly solved using deep learning. Different features are used as parameters for the models. For example, in fake news identification, Hessel and Lee (2019) explored the combination of different models and features, including hand-designed features, word embeddings, ratings, number of comments and structural aspects of discussion trees. In addition, another key element of the detectors is the datasets. For some threats (e.g., hate speech and fake news), few standard datasets target social media, but that is not the case for all threats. For violent content detection, for example, there is not a standard dataset focused on SM to the best of our knowledge. In order to overcome these limitations, works such as Bilinski and Bremond (2016) and Zhou et al. (2018) use a proxy dataset, such as Hockey Violence Dataset (Nievas et al., 2011).

Regarding the content analysis to extract users' affective state, beliefs and opinions, similar approaches are viable. Affective Computing aims to recognize, infer and interpret human emotions (Poria et al., 2017), distinguishing between sentiment analysis, polarity of content (e.g., Liu et al., 2017; Guo et al., 2018; Gupta et al., 2018), and recognition of the emotions present in a piece of information (e.g., Baziotis et al., 2018; Ahmad et al., 2020). In comparison, Opinion Extraction aims at discovering users' interests and their corresponding opinions (Wang et al., 2019). In general, the systems extract the entity or the target, the aspect of the entity, the opinion holder, the time when the opinion was expressed, and the opinion (Liu, 2012). Similarly, the positive aspects of social media interaction, crucial for estimating the CWB, could be extracted. Still, they have attracted less attention, but see Wang et al. (2014) and Chen et al. (2017).

Despite the success achieved by these efforts, the robustness of these systems is still limited. For instance, seldom they can generalize to new datasets and resist attacks (for example, word injection) (Hosseini et al., 2017; Gröndahl et al., 2018). An example of that is the case that occurred in the OffensEval shared task (Zampieri et al., 2019), where different hate speech classification models were compared in different subtasks. The best system in Subtask B (i.e., Han et al., 2019) ranked the 76th position in Subtask A that is a general and simple case of Subtask B.^4^ This example stresses how small changes in these tasks may drastically impact system performance informing on the challenge of applying these approaches in the dynamic contexts of social media. Some recent models can generalize the task while maintaining similar results in different platforms and languages under certain conditions (Wilkens and Ognibene, 2021b).

## 6. Use case

The following scenario is an example of how the Companion enables the personalization of educational interventions to help develop users' resilience against social media threats. The focus of this use case scenario is on the algorithmic threat of filter bubbles and how it can affect the users' perspective of healthiness. The content threat is associated with body image concerns (Marengo et al., 2018).

Alex is a 15-year-old high school student who spends a fair amount of his free time on his phone on a daily basis.

*Without the Companion:* Alex scrolls through his social network newsfeed and encounters a photo of an influencer that promotes masculinity. As summer is approaching, he decides to check the influencer's profile for possible tips to help him tone his body. Alex spends the next hour watching videos in the influencer's profile and starts following similar profiles. The social media platform algorithms learn that Alex is interested in posts related to masculinity, and he can spend hours interacting with this type of content. Thus, to maximize engagement, the platform starts displaying more content related to masculinity. Occasionally, the platform presents an advertisement in the form of a post to indulge Alex to buy a related product. Alex now finds his newsfeed to be filled up with fitness influencers and fitness products. Day by day, he likes and follows more fitness influencers, slowly leading his newsfeed to be full of fitness influencers that promote a specific body type. Through time, Alex's opinion regarding beauty standards starts to shift. He starts to believe that the male body needs to be muscular to be considered attractive and healthy. When looking in the mirror, he now feels that his body is far away from being considered attractive, and he will never be able to reach the beauty standards that have been set. He starts feeling unhappy with his body and seeks comfort through his social media platform. He comes across an influencer that promotes a product for rapid muscle growth and decides to look further into his profile. There he encounters photos that show a drastic change in the influencer's physical appearance claimed to be the result of the product. Alex decides that this product is the solution to his problem and buys it.

**Figure 4:**
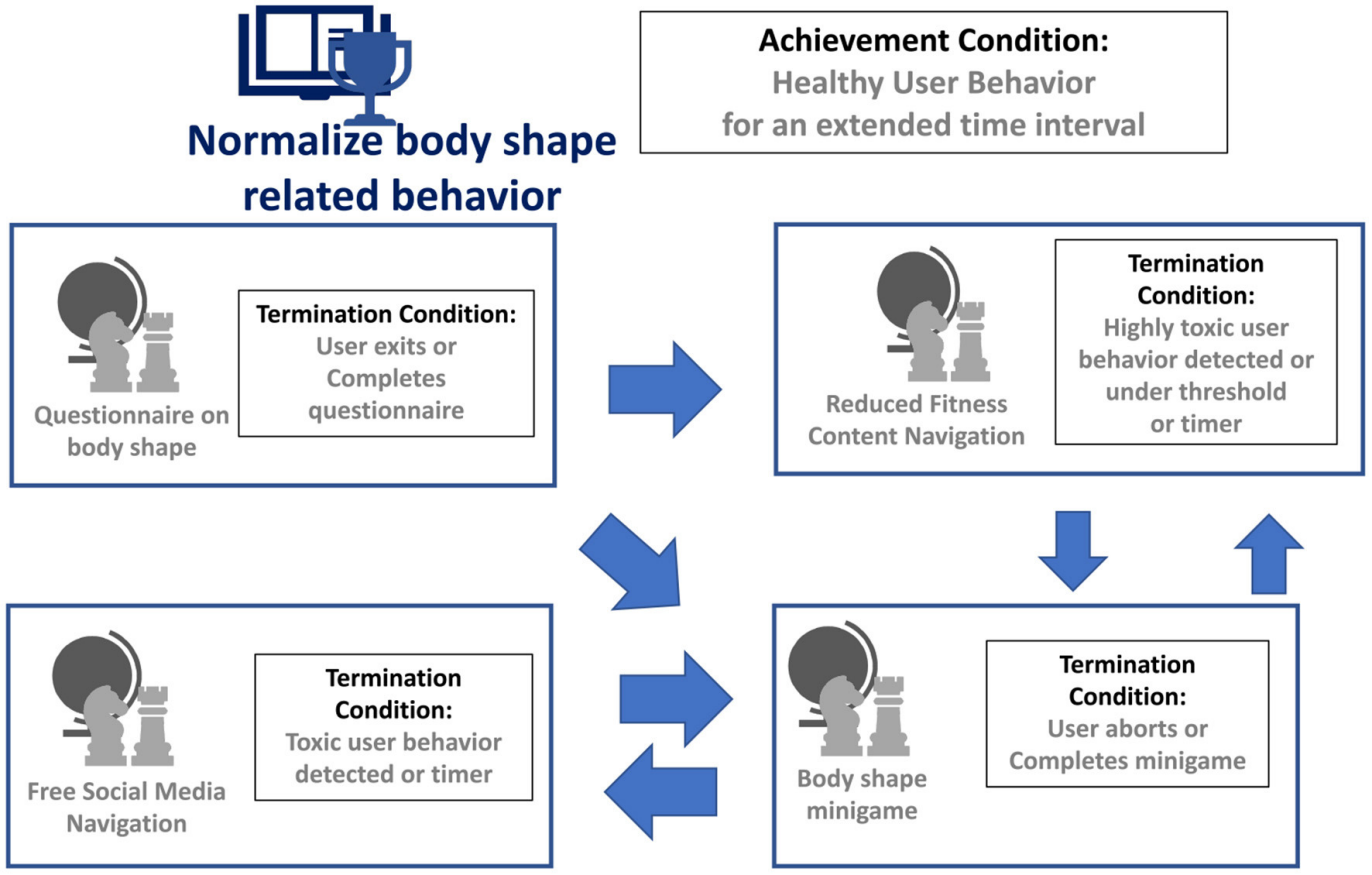
*Educational strategy example*. A visual example of how the policy to improve body shape-related behavior is accomplished within the platform. An initial questionnaire is completed by the user to determine if their behavior is classified as healthy or toxic. In the scenario that the questionnaire results come back as healthy, the user is placed into a free social media navigation state. This state will be terminated when the system detects that the user's behavior is no longer classified as healthy. This classification is done by analyzing the profiles the user has been following based on their category and further analyzing them with image classifiers. In this case, the system detects that the user's behavior has shifted from healthy to toxic a learning activity is initiated. The user is then placed into a state where the system alters the content they receive in their newsfeed.

**Figure 5:**
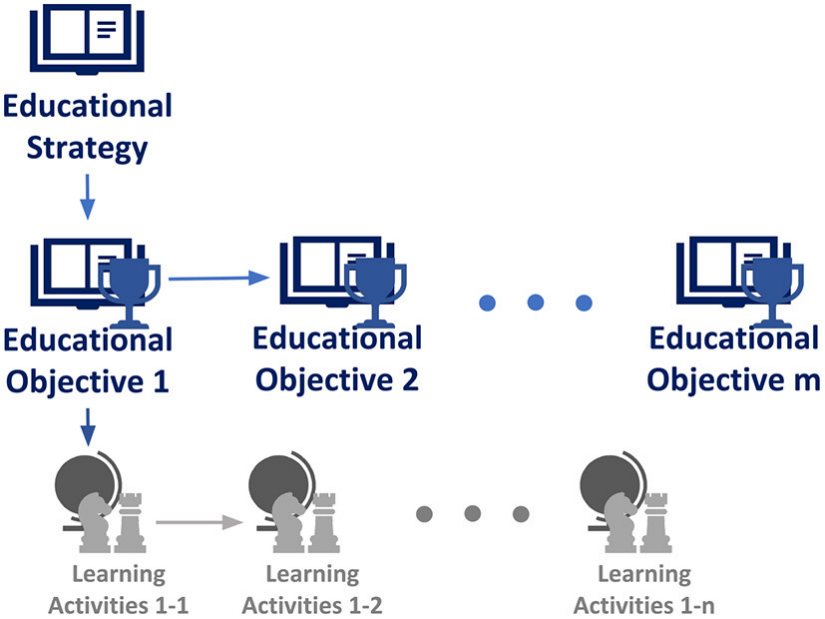
*A visualization of the hierarchical structure of the educational strategy*. Each educational strategy (narrative script) has a set of educational objectives that can be reached by a sequence of adaptive learning activities. The learning activities can be in the form of free-roaming, guided roaming, quizzes, minigames, or participating in group tasks. They are triggered based on the user's behavior within the platform.

*With the Companion:* Alex scrolls through his social network newsfeed and encounters a photo of an influencer that promotes masculinity. As summer is approaching, he decides to check the influencer's profile for possible tips to help him tone his body. Alex spends the next hour watching videos in the influencer's profile and starts following similar profiles. The Companion runs in the background and detects that the majority of profiles Alex has started to follow fall under the category of fitness. Image classifiers further identify that those profiles promote a specific body type. Then, the Companion triggers a narrative script and notifies Alex that a new game (the script) is available (Figure 4). Alex accesses the game and initiates the narrative script. The narrative script mechanisms assign him to an influencer that supports the opposite perspective (counter-narrative) than the one he triggered. He is instructed to navigate through the profile and self-reflect on how this profile makes him feel. Alex is asked to participate in an online collaborative game showing the impact of social media influence and filter bubbles on our decision capabilities (e.g., see Lomonaco et al., 2022b and Section 7.1). He is then shown a brief video of how SM algorithms work and how they can place a user into filter bubbles. In the next screen, Alex enters a mini-game where he is instructed to manipulate a filter bubble by following and unfollowing profiles and by liking and unliking posts. During the game, Alex can see how the newsfeed of the user changes according to his behavior. Alex starts to understand how social media works and how algorithms can learn from our behavior. Once the game is over, the narrative script ends, and Alex receives a badge for completing it. The educational component registers Alex's signs of progress and marks the learning objective of filter bubbles as complete (Figure 5). Alex returns to his social media profile and receives a notification from the Companion that the content of his newsfeed has been altered by the CWB-RS component to reduce the harmful content that he has been receiving. He has the option to revert this setting, but he decides to continue with it. The CWB-RS component filters Alex's news feed with images unrelated to muscular fitness. Eventually, this alters Alex's content needs and influences him to start following profiles that are not solely related to muscular fitness, which leads to minimizing his exposure to influencers promoting a perfect body. To confirm that Alex is staying on the right track, a few days later, the Companion operates a further inspection to analyze the content being followed. The Companion verifies that Alex's online behavior has improved after the completion of the mini-game and it does not trigger any further mini-games for him. Alex receives a notification informing him that the CWB-RS component has stopped altering his newsfeed. His newsfeed content has now become more balanced. Alex has become less obsessed with the idea of having a muscular body.

## 7. Preliminary experimental results

The realization of the COURAGE companion is progressing through the study of different educational strategies and the development and testing of educational tools and computational components.

### 7.1. Educational and psychological studies

Educational and psychological studies are the starting point to define objectives, methodologies, and tools that will be integrated into the Companion and guide the participatory design of the *educationally managed social media community*.

Data was collected through online studies to calculate correlations between toxic content tagging (e.g., disagreement measure) and personality traits (e.g., cognitive empathy or authoritarianism). A link between learners' judgments and personality traits could only be weakly found for authoritarianism (Aprin et al., 2022a). We also studied users' intentions to share emotional images. The study was conducted in Italy and Germany, with university students surveyed online in Germany. The evaluation of nearly 200 students is not yet completed. It is expected that results will provide insights into the relationships between socio-emotional competencies, moral values, and the willingness to share images with diverse social groups.

Similarly, a pilot study aimed to investigate the relationships between emotional intelligence and social media threats was conducted involving 110 adolescents of a secondary school in Italy during an extracurricular school activity (Scifo et al., 2022). In particular, two research studies have been conducted within this pilot. The first study had the purpose of investigating the relationships between emotional intelligence and adolescents' ability to detect fake news on social media. The second study included a training path aimed at stimulating emotional intelligence and promoting a conscious use of social media. Moreover, the training path has also contributed to raising adolescents' awareness of bullying and cyberbullying. The analysis of the results is ongoing. These studies will drive the development of new educational components for the companion and help to define the companion's personalized educational strategies.

We tested a game-based educational experience (De Gloria et al., 2014) to increase students' awareness of social media algorithmic threats, focusing on filter bubbles and echo chambers inspired by the “wisdom of crowds” (Lorenz et al., 2011; Becker et al., 2017). It was tested with both University and High school students providing encouraging results (Lomonaco et al., 2022b). While more data is being collected a specific component is being designed to reproduce the experience inside the companion.

Furthermore, we developed a scenario to inform students about racist content on social media. Here, users are informed about the background of racism by the virtual companion in a closed social media environment. By means of the results of an experimental study in which the virtual learning companion either transmits information on racism (experimental group) or not (control group), we will analyze the effects on users' knowledge and awareness regarding racism.

Also, we are constantly working on a scenario for empathy training which shall sensitize young users regarding the negative effects of cyberbullying. For this training, for instance, a video showing an example and providing a definition of empathy will be shown to students in an experimental study in Germany and Spain. We hypothesize that students who completed the empathy training will be more sensitive to cyberbullying and are less likely to intend to bully in the future.

Finally, in Taibi et al. (2021) we present a platform specifically designed to support the development of competences related to Information and Data Literacy. This platform extends the open-source alternative to Instagram called Pixelfed, with functionalities designed to support students in increasing their awareness of social media mechanisms based upon artificial intelligence algorithms. A pilot with secondary school students has been conducted to experiment educational activities based on the proposed platform.

### 7.2. Educational components

Several components and applications, that will later be integrated in the Companion, are being developed and tested.

A number of mini-games to increase social media awareness, covering topics such as the digital footprint, social media addiction, misinformation and body image dissatisfaction have been designed and tested. For instance, the serious mini-game “SwipeIt” for sensitizing students to toxic content (e.g., cyberbullying), was endowed with additional features like a multi-language interface.

One of the scenarios using the Companion aims at raising learners' awareness of fake content in an Instagram-like social media environment (Aprin et al., 2022a). The VLC guides the learners through various examples in a chatbot-like dialogue. Additionally, learners are provided with access to other instances of the embedded images that are found through Google Reverse Image Search. The idea is that seeing the image in other contexts provides clues for judging the credibility of the presented content. The Companion in this scenario has been implemented as a Chrome browser plugin, which allows for running the scenario in a familiar web environment. Initial tests with a heterogeneous group of users indicated that the environment is perceived as supportive and usable for the classification task. Subsequently, the scenario has been tested in a secondary school classroom setting with 30 students. Preliminary findings suggest that the Companion was effective in supporting the decision about the veracity of the images shown.

The Narrative Scripts for empowering digital and self-protection skills of users through the use of computer-supported collaborative learning activities and the help of a virtual companion were presented at the Sixteenth European Conference on Technology Enhanced Learning in Italy (Hernández-Leo et al., 2021). Over 200 school workshops were conducted involving over 1.000 adolescents in private and public schools in Barcelona. Simultaneously, a light version of school workshops and the study was replicated at the University of Campo Grande (Brazil). These workshops contributed to the testing and the fine-tuning of the educational tools developed by UPF. Data collection included information derived from the implementation of Narrative Scripts, PyramidApp and EthicsApp, based on the studies Collaborative Learning for digital Environment (CSCL), Sequencing in Learning, and the evaluation of Narrative Scripts to raise teens' social media awareness.

Most workshops have been media education interventions with Narrative Scripts. The final result consisted of a social media simulated environment supported in Pixelfed. The pilots consisted of a six-module intervention with teaching and learning activities supported by Narrative Scripts and other gamifying elements. The interventions were diversified to integrate interactive features supported by AI elements, image decorations and “smart narratives” (allocation of roles/counternarratives; decorations in shared content). The first results of the data collected evaluate how the adaptive educational intervention embedded in the Narrative Scripts facilitates a suitable approach to educating adolescents about body image and stereotyping in social media. In particular, the analysis examines and compares approaches to identify the dominant body image stereotype in students' social media. Results showed that the use of xAPI (tracking user behavior in Pixelfed) combined with self-reported answers can provide a satisfactory detection of adolescents' educational needs, so as to enable automatic distribution of suitable counter-narratives (out of a collection) to students in the scripts (Lobo et al., 2022).

We also developed a visual interface that augments tweets with machine learning-based detectors of different forms of toxic content. To help the interpretation of this information created by state-of-the-art components, a web page was built showing correct and erroneous results produced by the detectors on different types of content, that will soon be tested in educational activities in high schools.

### 7.3. Computational components

The computational backbone of the Companion, which comprises diverse components such as content popularity predictors, user models and recommenders, is being developed, tested and outlined in Aprin et al. (2022b). Particular effort has been devoted to the development of content-based threat detectors because of their multiple roles: a) triggering specific educational activity, b) evaluating community well-being, and c) supporting recommendation and re-ranking of content.

Models to detect fake news and irony were presented at LREC 2022 (Hartl and Kruschwitz, 2022; Turban and Kruschwitz, 2022). The fake news detection system has established a new-state-of-the-art benchmark performance on the commonly used FakeNewsNet dataset. To improve performance and find the best trade-off with computational cost, the detectors were continuously updated and different architectural patterns (e.g., graph neural networks) were explored. The results were presented at several competitions about fake news detection as well as topics around hate speech (Wilkens and Ognibene, 2021a,b; Lomonaco et al., 2022a), organized within the scope of well-established annual events such as CLEF 2021, CLEF 2022 and GermEval 2021. Although submissions were very competitive, the contributions by UR resulted in winning the German cross-lingual fake news detection challenge at CLEF 2022 “CheckThat!” (Tran and Kruschwitz, 2022) and being runner-up in the fact-claiming comment identification at GermEval 2021 (Tran and Kruschwitz, 2021).

**Figure 6:**
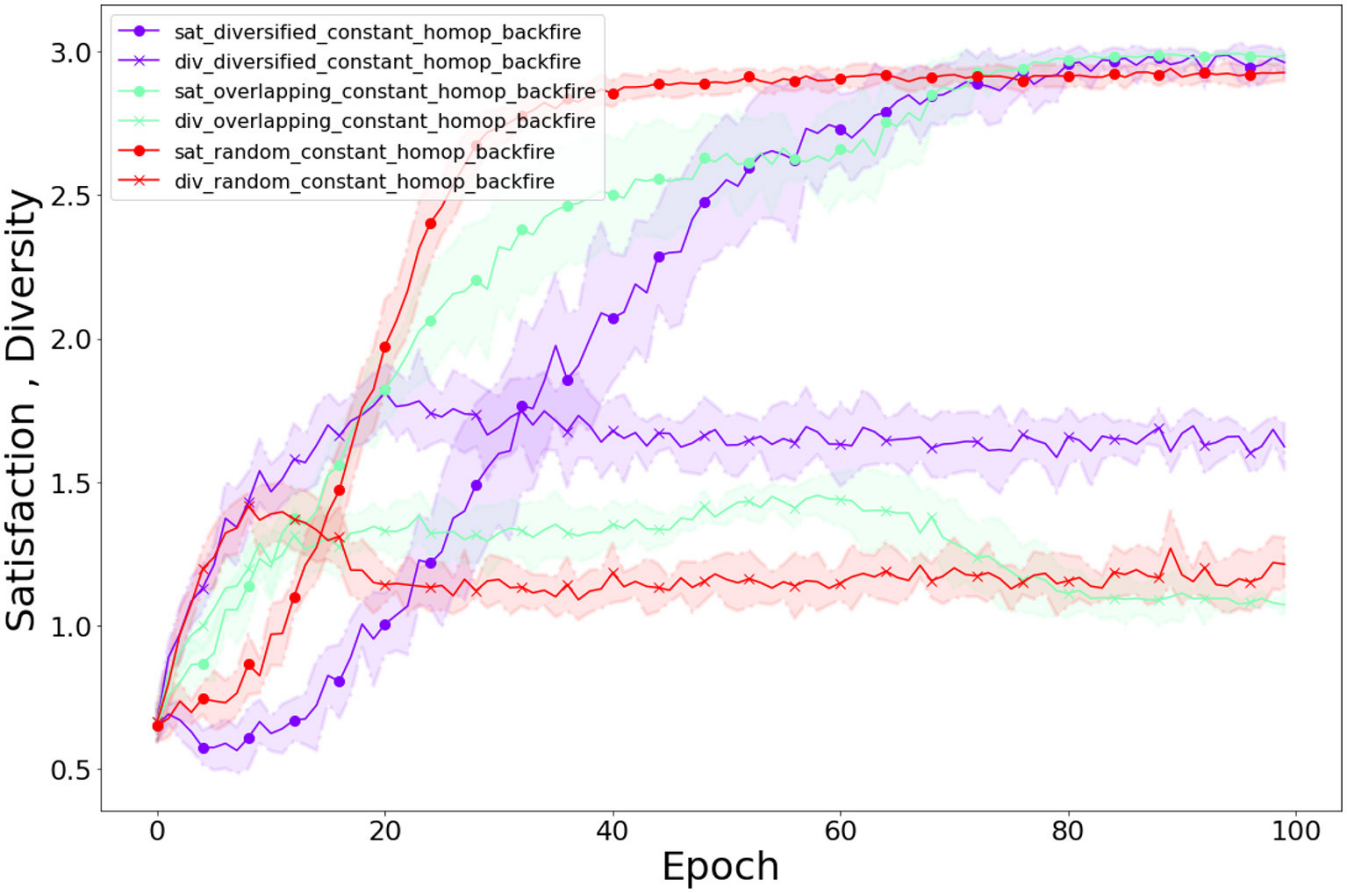
*Simulated impact of different recommender systems on users' satisfaction and content diversity exposure*. Satisfaction is assumed as a proxy for the sustainability of the social media platform. Content diversity exposure could play an important role in countering the effects of filter bubbles (Bozdag and van den Hoven, 2015; Nikolov et al., 2015), echo chambers (Wolfowicz, 2015; Bessi, 2016; Gillani et al., 2018), and ultimately society polarization. Mean and standard deviation over 10 runs with three different new connections *Recommenders*: maximize opinion diversity, random, overlapping neighborhood. For each strategy (colors) Satisfaction (“o”) and diversity (“x”) are pictured. *Overlapping*: recommend users with the highest number of common friends. *Diversified*: recommend users with the highest opinion difference. *Random*: baseline, recommend random users. *Satisfaction*: the mean distance for each user between his opinion and the ones in his feed. *Diversity*: entropy of binned opinions that populate users' feed in each time step. Highlighted areas represent standard deviation across different runs. Each social network is initialized with 100 users (nodes) and connections (edges) are created with an adaption of preferential attachments (Albert and Barabási, 2002). Differently from Albert and Barabási (2002) the nodes' probability of being connected with an incoming node is not proportionally related to nodes' degree but is related with their opinion distance. Users were modeled by extending the model proposed in Geschke et al. (2019) with a backfiring component (Bail et al., 2018), i.e., users exposed to opinions that were distant from theirs moved in the opposite direction. The recommender that maximizes diversity between the pairs of users to connect showed a slower start but achieved higher exposition to more diverse content and a similar level of satisfaction to the other two RSs.

Finally, we experimented with different models of social network connectivity and user behavior. Several computational experiments showed that recommender systems have a substantial impact on the user experience on social media. For example, we simulated the impact of different recommender systems on combinations of users' satisfaction and content diversity exposure as proxies of potential components of the CWB metrics. Satisfaction is assumed as a proxy for the sustainability of the social media platform. Content diversity exposure could play an important role in countering the effects of filter bubbles (Bozdag and van den Hoven, 2015; Nikolov et al., 2015), echo chambers (Wolfowicz, 2015; Bessi, 2016; Gillani et al., 2018), and ultimately society polarization (Cinus et al., 2022). In the results shown in Figure 6. We compare three different new connection recommenders: maximize opinion diversity, random, overlapping third order neighborhood. Users were modeled by extending the model proposed in Geschke et al. (2019) with a backfiring component (Bail et al., 2018), i.e., users exposed to content presenting opinions distant from theirs changed their minds in the opposite direction. The recommender that maximizes the diversity of opinion between the pairs of users to connect showed a slower start but achieved higher exposition to more diverse content and a similar level of satisfaction to the other two RSs. In the near future, we aim at integrating a full CWB-RS with educational objectives in the simulation.

## 8. Discussion and conclusion

This contribution is motivated by the desire to improve the impact of social media on our society. They have indeed several positive effects (Wang et al., 2014; Chen et al., 2017): they extend our capacity to be connected with our contacts, create new useful social connections, and scale up and accelerate social interactions. Moreover, they supported various forms of activism (Gretzel, 2017; Murphy et al., 2017) and even enabled whistle-blowing in oppressive regimes (Joseph, 2012) as well as protests organization (Gladwell, 2011; Shirky, 2011). However, what can be defined as an explosion of SM has also brought several new negative social phenomena, such as digital addiction (Kuss and Griffiths, 2011; Young, 2017) and exacerbated existing ones, e.g., misinformation (wildfires) (Webb et al., 2016), which existed only on a limited scale and slow pace before.

Teenagers are a group that is particularly affected by numerous social media threats (Clarke, 2009; Ozimek et al., 2017). We propose an educational and support platform, a Companion, focused on rising teenagers' “new media literacy” (Scolari et al., 2018), “digital citizenship” (Jones and Mitchell, 2016; Xu et al., 2019), and awareness of social media threats. The Companion will allow the smooth passage from everyday life use of social media to an educational experience by interfacing with the students to support and guide their interaction with the social media environment both inside and outside the classroom. Several components of the Companion have been developed and successfully tested, as briefly described in Section 7.2.

In social media communities, as in any society, the safety and well-being of its members are determined by their own mutual interactions (Jones and Mitchell, 2016). Therefore, an important endeavor is to increase users' awareness of the consequences of their actions and acceptance of necessary boundaries, especially in such deindividuating environments (Lowry et al., 2016). The presence of a trade-off between users' rights and duties or freedom VS safety introduces ethical issues (EUC, 2019; Ienca and Vayena, 2020) (e.g., defining what is considered hate speech) that require the formulation of a comprehensive and shared view of the values of the social media community. This led to the introduction of the concept of Collective Well-Being (CWB) for Social Media communities, the shared view of the desirability of the conditions of the specific community, which would drive the definition of the educational objectives and the desired behaviors of the community members. To define the desired social media community as well as the corresponding CWB objectives, the explicit community regulations, and the educational objectives necessary to support them, we argued for a collaborative participatory design approach involving experts, educators, and community members, i.e., parents and teenagers (Sánchez-Reina et al., 2022).

In Section 4.3 a methodology is proposed to measure from online behavior the CWB of social media communities. Defining an operational measure of CWB could help deal with the cognitive and algorithmic threats that characterize social media and may hinder the effectiveness of purely educational efforts. A CWB measure could help transfer the community interests and values, as well as the educational objectives, to the recommendation algorithms that drive the users' experience by selecting and ordering feeds and connections. In the Companion this will be realized by the Collective Well-Being Recommender System (CWB-RS), which sequences educational activities and balances the content presented to the students in order to maximize the CWB (see Section 5).

From a technical point of view, the problems are multiple. Starting from the formulation of the CWB measure, the number of aspects to balance and the likely non-linear interactions between the single and the community sub-groups will require an iterative design approach. Moreover, while the state of the art for the components that detect the relevant quantities is constantly improving (e.g., sharing of hate speech in the community, see Table 3 and Sections 7.3, 5.4), the process is still noisy. The development of active evaluation methodologies, possibly involving educators as humans-in-the-loop, is a possible way forward. The CWB-RS must face additional complexities to evaluate the longer-term impact of its recommendations for the achievement of educational objectives and the future CWB of the community as well as balancing the level of engagement necessary for the educational and social functions (e.g., finding out that a friend needs online support) while avoiding digital addiction. We discussed in Section 5 that these issue may require combining an intelligent tutor system with recommender systems built using the hierarchical reinforcement learning framework.

Our contribution in this paper, in particular our experimental studies, are specifically designed for relatively small communities that can tailor the approach to their own specific needs. One may be tempted to think about scaling up the whole approach and integrating the educator in the loop, the CWB and CWBRS on the global social media platforms. This would prohibitively escalate the moderation costs that are already very demanding (Steiger et al., 2021) and would have to take also the educational aspect into account, which requires wider expertise and user-specific policies. From an ethical point of view, the undertaking would be enormous. While privacy, censorship, freedom of speech, misinformation campaigns and hate speech are strongly involved ethical problems, they are by now very common in the discussion about social media (Webb et al., 2016), especially after Twitter permanently banned Donald Trump (Courty, 2021). However, the formulation of a CWB for social media requires not only formulating a metric that balances many different demands but it justifying the worldwide and cross-cultural adoption of a value set that supports such a metric applied to the social and dynamic version of the *WWW*.

Currently, the international community is undertaking a substantial effort in understanding and regulate the ethical implication of AI systems (EUC, 2019; IEEE, 2019; OCED, 2019; UNESCO, 2020). Unmistakably there are mixed ethical and technical issues that go beyond those currently faced (EUC, 2019; IEEE, 2019; OCED, 2019; UNESCO, 2020).^5^ For example, trying to optimize the CWB may induce a further increase in social media complexity. This may reduce even more our control over social dynamics (Floridi, 2014) and backfire with even more threatening, addictive, and unhealthy dystopian situations.

While it is crucial that the international community continues its effort and targets social media (Gorwa, 2019), we highlighted that aiming to improve the CWB of SM local communities implies first and foremost aiming to educate local communities themselves, as the CWB depends on users' attitude, interactions and relationships (Jones and Mitchell, 2016). Education is the best way we know to improve human behavior. Indeed, if the methodology is successfully applied on a sufficient scale improving the members' new media literacy and digital citizenship, it may improve the general impact of social media on our society. Focusing on more controlled communities, e.g., schools, with a very limited scale for the social media domain reduces the ethical burden on the design side as well as the technical demands for accuracy and reliability through the integration of a mediator role for educators and parents, through a “Human in the Loop” paradigm. This approach also allows focusing on the critical educational aspect. The creation of educationally managed social media communities allows supported learning experiences and a full range of new experiments (Amarasinghe et al., 2021; Fulantelli et al., 2021; Hernández-Leo et al., 2021; Malzahn et al., 2021).

Differently from previous other interventions with a similar aim, this paradigm enabled by the educational virtual Companion for social media has indeed the potential to provide an educational experience on a scale comparable to that of the social media platforms. Indeed, it will be challenging to define an educational path that covers most of the numerous points of interest in digital citizenship (Jones and Mitchell, 2016; Xu et al., 2019). Also, the integration of this educational experience in student life is challenging, especially regarding the experience outside the classroom, where the non-educational global platforms will compete for student time and attention. Still, we believe that the combined technological and educational strategy implemented by the Companion has a good chances to be effective in containing many of the current social media threats.

Finally, this approach is the perfect means to bootstrap and test the concept of CWB-RS systems, verify their feasibility, stability and robustness, and create suitable datasets. The data collected from this initiative may not only be useful for replicating and extending this type of educational approach, but it could also be a first step to provide evidence that social media's impact on society can be improved by taking the community needs more into account in their design. A characteristic feature of social media is that they are crucially the result of a community activity, which both consumes and produces their content. It is daunting that platforms' objectives are so detached from those of the community. Therefore, we hope that our results can support the process of introducing new evidence-based regulations both for the platforms and their algorithms, beginning with requesting the platforms to release their data for scientific research and enable large-scale studies, which have been curbed after the limits they recently set following Cambridge Analytica and other scandals (Hemsley, 2019).

## Data availability statement

The original contributions presented in the study are included in the article/supplementary material, further inquiries can be directed to the corresponding author.

## Author contributions

DO conceived of the presented ideas and concepts and contributed to the design of the educational platform architecture. RW and GD supported, revised the design, and developed the content machine learning aspects. UK proposed, described the integration of behavioral economics methodologies for education, and supervised the information retrieval aspect of the contribution. FL and SB helped with the aspects of network dynamics and dataset collection. DH-L, JS-R, ET, and RL contributed with the proposal of playful educational methodology, the cooperative social media design, and narrative scripts. UH, NM, FA, VS, JB, and SE overviewed the conceptual development. DT contributed to the design of the educational platform architecture, DT and LS contributed with expertise on digital addiction and digital literacy intervention design. All authors contributed to the as article and approved the submitted version.

## Footnotes

^1^Simon Wiesenthal Center: http://www.digitalhate.net, Online Hate and Harassment Report: The American Experience 2020: https://www.adl.org/online-hate-2020.

^2^Retrieved from here: https://www.bbc.co.uk/programmes/articles/4fRwvHcfr5hYMMltFqvP6qF/help-your-students-spot-false-news BBC, (UK), https://literacytrust.org.uk/programmes/news-wise/ NewsWise (UK).

^3^Retrieved from: https://voxeu.org/article/defence-gdp-measure-wellbeing.

^4^We highlight that, despite this extreme case, systems tended to maintain a similar performance across the different subtasks.

^5^See https://standards.ieee.org/standard/7010-2020.html IEEE 7010-2020 - IEEE Recommended Practice for Assessing the Impact of Autonomous and Intelligent Systems on Human Well-Being.
